# All-Solid-State Ion-Selective Electrodes with Multi-Walled Carbon Nanotubes Based on Glassy Carbon for Biguanide Derivative (Chlorhexidine Digluconate)

**DOI:** 10.3390/s26206633

**Published:** 2026-10-20

**Authors:** Joanna Lenik, Katarzyna Witkowska

**Affiliations:** Department of Analytical Chemistry, Institute of Chemical Sciences, Faculty of Chemistry, Maria Curie-Sklodowska University, Maria Curie-Sklodowska Square 3, 20-031 Lublin, Poland; kasiawitkowska02@gmail.com

**Keywords:** biguanide derivative, chlorhexidine, solid contact electrode, multi-walled carbon nanotubes, potentiometry

## Abstract

This paper presents research on the properties of solid contact electrodes with an intermediate layer of multi-walled carbon nanotubes. They were used as an intermediate layer (Solid Contact SC-ISE electrodes) and as a dispersed material in a polymer membrane (Single Piece SP-ISE electrodes). The properties of neutral macrocyclic compounds as active ion-sensitive substances in membranes, in which plasticizers with varying lipophilicity were also used, were compared. The selected optimal sensor shows a theoretical Nernstian response for the divalent chlorhexidine ion (z = 2) of approximately 29 mV decade^−1^ over the linearity range of 10^−6^–10^−3^ mol L^−1^. A short response time of approx. 10 s, potential stability (the potential drift is several dozen μV min^−1^ in a 10^−4^ mol L^−1^ chlorhexidine solution over 4 h), and high potential reversibility (the standard deviation in a 10^−5^ mol L^−1^ solution is 0.69 mV, whereas in a 10^−4^ mol L^−1^ solution it is 0.71 mV) are further advantages and benefits of the developed modern electrodes. In general, it can be concluded that the SC-ISE showed better potential reversibility than the SP-ISE, particularly at chlorhexidine concentrations of 10^−4^ and 10^−5^ mol L^−1^, while also exhibiting improved resistance to water-layer formation. High selectivity was also achieved in relation to ionic substances (sodium, potassium, calcium, magnesium, acesulfame ions, and neutral substances used as excipients in chlorhexidine preparations, i.e., d-mannitol, d-sorbitol, sucralose, propylene glycol, xylitol, aspartame). The sensor with satisfactory parameters was used to determine the API content in the pharmaceutical preparation, Eludril, with a recovery of 95.9–98.6% and a relative standard deviation (RSD) ranging from 0.7 to 3.7%.

## 1. Introduction

Chlorhexidine (CHX) is a cationic compound of the bisbiguanide group, widely used as an antiseptic and disinfectant due to its broad-spectrum antibacterial activity. It is regarded as one of the most effective compounds for controlling microorganisms, as it does not induce significant resistance in various microbial strains. The biochemical mechanism of action of CHX involves irreversible damage to microbial cytoplasmic membranes [1,2].

From a chemistry point of view, the chlorhexidine molecule contains two 4-chlorophenol rings linked by two biquanidine groups and a hexamethylene chain. Chlorhexidine is insoluble in water; it is only after the protonation of the secondary amine groups (-NH-) to acids that soluble salts such as digluconates, dioctanes, or hydrochlorides are obtained. According to the literature, the pKa values of chlorhexidine are not consistent, as they depend on the method of determination and on which protonation equilibria are taken into account. According to [3], the apparent pKa values of chlorhexidine have been reported as approximately 2.2 and 10.3, and the molecule exists predominantly in a dicationic form over the pH range relevant to aqueous and physiological conditions. Another reference [4] indicates that pKa_1_ = 10.3 is associated with the first two stages of protonation, while the next value, pKa_2_ = 2.2, is associated with subsequent protonation stages, and within the pH range 4–8, chlorhexidine is a dication. The predominant dicationic form of chlorhexidine in the studied pH range (pH 4–8) is consistent with the theoretical Nernstian slope of approximately 30 mV decade^−1^ expected for a divalent ion.

Chlorhexidine is used in many pharmaceutical preparations, including those for oral hygiene, such as aqueous solutions and mouthwashes, where its concentration must be carefully controlled to ensure the product has the desired effect [5,6]. Several analytical methods have been developed for the quantitative determination of chlorhexidine in pharmaceutical preparations, including chromatographic, spectrophotometric, electrophoretic and electrochemical methods [7]. Among these methods, potentiometric methods based on ion-selective electrodes are particularly attractive as they enable the direct, accurate and precise measurement of ions using relatively simple, inexpensive equipment and without the need for complicated sample preparation.

Solid-contact ion-selective electrodes (SC) are currently one of the most rapidly developing groups of potentiometric sensors. In these electrodes, the function of the Ag/AgCl, Cl^−^ redox system—which involves the exchange of electrical charge and ensures ionic and electronic conductivity—has been taken over by other solid materials, which are referred to as the solid contact. Electrodes without an internal solution, so-called SC-ISEs, have many advantages, including the following: a reduction in the limit of detection through the elimination of release and the concentration gradient of the major ion in the membrane [8,9], an easier-to-modify design, simpler and cheaper preparation due to their small size, and easier transport and storage. Another very important advantage is their greater mechanical strength, owing to the greater membrane thickness and the absence of the frequently occurring phenomenon of air entrapment in the internal solution during electrode use [10].

In a subsequent stage of the research aimed at developing solutions that would reduce potential instability and facilitate contact between the electron and ion conductors, intermediate layers were introduced. This led to the establishment of various types of all-solid-state electrode designs, which include the following electrodes: SC-ISE (solid contact ISE), SP-ISE (single-piece ISE), and CP-ISE (conducting polymer ISE). In the case of SC-ISE electrodes, the solid contact performs only the function of a mediating layer, which is placed between the conductive surface and the ion-selective membrane. In the next design, the SP-ISE, the solid contact material was incorporated directly into the membrane phase. In contrast, in the final type of electrode, the CP-ISE, the conductive polymer acts as both the mediating layer and the receptor layer [11,12,13,14]. The introduction of a mediating layer of solid contact in the design of ion-selective electrodes has led to an improvement in the reversibility and stability of ion-electron conductivity, an increase in the charge transfer current at equilibrium—eliminating the possibility of system polarisation—and the elimination of side reactions other than the main electrode reaction [15]. Solid contact materials should, in addition to reversible ion-electron conductivity, provide high redox capacity or volumetric capacity in order to ensure potential stability. They should be highly hydrophobic to prevent the formation of a water layer between the solid surface and the membrane, chemically stable (no side reactions), and also mechanically durable and resistant [16,17].

Materials exhibiting mixed ionic and electronic conductivity include conductive polymers, in which double bonds provide electronic conductivity, whilst doped ions provide ionic conductivity. The interfacial potential is controlled by a redox reaction, as a result of which the entire system exhibits a high redox capacity, which results in high stability of the sensor potential [18,19,20]. Conductive polymers that are used as solid contacts include: polypyrrole (PPy), poly(3,4-ethylenedioxythiophene) (PEDOT), poly(3-octylthiophene) (POT), polyaniline (PANI) [21,22] and poly(N-methylpyrrole) (PNMP) [23]. In addition to the above-mentioned compounds used as solid contacts, other materials have also been introduced, such as self-organizing redox layers [24], hydrogels with immobilized internal electrolytes [25], or ionic liquids [26].

Another group of compounds that, unlike conductive polymers, exhibit ion-electron conductivity through the formation of an electric double layer at the phase boundary between the membrane and the solid electrode substrate is nanomaterials. These include: carbon nanomaterials, i.e., fullerenes, carbon nanotubes, graphene, three-dimensionally ordered macroporous carbon (3DOM), mesoporous colloidal carbon (CIM), carbon black, and others, such as metal nanoparticles of gold, platinum, and molybdenum sulfide [22,27,28,29].

Multi-walled carbon nanotubes are particularly interesting due to their favorable properties and the wide range of applications in ion-selective electrodes. Carbon nanotubes are characterized by unique chemical, mechanical, magnetic, electronic, and optical properties. Their chemical properties include high resistance to a variety of chemical compounds and many environmental conditions, and they are stable over a wide pH range. Their high mechanical strength stems from the presence of very strong C–C bonds within the CNT structure. Another advantage is their high specific surface area, resulting from their cylindrical, nanometric structure. This facilitates efficient charge exchange with the environment and enables integration with other functional materials. Their hydrophobicity is also a significant feature, reducing the risk of a water layer forming at the membrane-solid interface. In the case of ion-selective electrodes, this property is crucial for the long-term stability of the potential [30,31].

In recent years, there have been many new and interesting reports on electrodes containing carbon nanotubes and carbon nanocomposites, particularly for the detection of drugs in biological or pharmaceutical samples [32]. An electrode sensitive to acetylcholine, which acts as a neurotransmitter in the human body, has been developed [33]. In the design of this sensor, a biomimetic composite material was used for the first time to act as an ionophore. This material was prepared by a polymerization method, which involved placing acetylcholine molecules within a pre-formed composite matrix of multi-walled carbon nanotubes and polyaniline. This composite, together with an ion exchanger dissolved in a mixture of poly(vinyl chloride) and a plasticizer, formed the ion-selective membrane of the electrode. Another electrode example is the potentiometric sensor developed for the determination of bambuterol hydrochloride [34]. The authors utilized, amongst other things, a carbon paste-based electrode containing composites of layered Mg-Al double hydroxides and multi-walled carbon nanotubes. In other studies by the same authors, the properties of ibuprofen electrodes with glassy carbon (GCE) were determined. Composites of multi-walled carbon nanotubes and β-cyclodextrin derivatives were used as the active substance in the polymer membrane [35].

Other interesting works published in recent years that have applied the all-solid-state electrode design with a carbon nanotube interlayer include the potentiometric determination of orphenadrine citrate [36], xipamide [37], tulathromycin [38], and gefitinib [39]. The latest literature reports also provide many other design options, including composite materials of conductive polymers and carbon nanomaterials, as well as metal oxides, as summarized in the review article [40] and in the paper [41].

Previous studies have demonstrated the feasibility of potentiometric determination of chlorhexidine using polymeric ion-selective membranes with different membrane compositions [42,43,44]. These studies focused primarily on the effect of membrane components on the performance of chlorhexidine-selective electrodes. However, the development of a fully solid-state design presents an additional design challenge concerning the efficient and stable conversion of ionic activity into an electronic signal. In this context, the use of multi-walled carbon nanotubes as an ion-to-electron transducer represents an attractive strategy for combining a chlorhexidine-selective membrane with a glassy carbon surface (GC/MWCNT). According to the literature [42,43,44], no chlorhexidine-sensitive ion-selective electrodes using multi-walled carbon nanotubes as a solid contact element have been described so far. The research described in this paper involves the characterization of electrodes with three different designs: ISE, SP-ISE, and SC-ISE. The best electrode obtained was the one with MWCNT as an intermediate layer with the HSB*β*CD + DDP + KTpClBP membrane composition and the following specified parameters: slope 29.4 mV decade^−1^, linear range 10^−6^–10^−3^ mol L^−1^, and LOD 6.3 10^−7^ mol L^−1^. The developed electrode exhibited high selectivity toward the tested matrix components, with negligible interference from non-ionic excipients such as d-sorbitol, d-mannitol, aspartame, xylitol, and sucralose, which supports its applicability to the analysis of chlorhexidine-containing pharmaceutical formulations. The electrode was used to determine CHD in the Eludril mouthwash.

## 2. Materials and Methods

### 2.1. Reagents, Apparatus

2-nitrophenyloctyl ether (*o*-NPOE) (Fluka, St. Gallen, Switzerland), bis(2-ethylheksyl)sebacate (DOS) (Merck Schuchard Hohenbrunn, Germany), diisodecyl phthalate (DDP) (Pftaltz&Bauer Waterbury, CT, USA), heptakis (2,3,6-tri-O-benzoyl)-*β*-cyclodextrin (HSB*β*CD), (dibenzo-18-crown-6 (DBC-6), potassium tetrakis(4-chlorophenyl)borate (KTpClBP) (Sigma-Aldrich, St. Louis, MO, USA), tetrahydrofuran (THF) (POCh, Gliwice, Poland), poly(vinyl chloride) high molecular weight (PVC), and magnesium ionophore I ETH 1117 (Sigma Aldrich) were the membrane reagents. The following other reagents were applied: a chlorhexidine digluconate (CHD) solution (20% *w*/*v*) (Alfa Aesar ThermoFisher GmbH, Braunschweig, Germany) and multi-walled carbon nanotubes (MWCNTs) with 6–9 nm diameter, 5 μm length, and 95% purity (Aldrich). All other chemicals were obtained in analytical reagent grade and also used as received. All aqueous solutions were prepared with deionized water with a conductivity of 0.07 μs/cm (Elix Advantage System Mili-Q plus Milipore, Spittal an der Drau, Austria). The pharmaceutical product Eludril (chlorhexidine digluconate) 0.2% *w*/*v* liquid mouthwash (Pierre Fabre, Castres, France) was used. The measurements of the electromotive force of the chlorhexidine electrode—the reference electrode (Orion 90-02) system—were made at 23 ± 1 °C using the Electrochemistry EMF Interface system (Lawson Labs. Inc., Malvern, PA, USA) and an IBM PC. The MWCNT suspension in THF was homogenized for approximately 10 min using a Bandelin SONOREX SUPER RK 100 H ultrasonic bath (35 kHz, 80 W) prior to drop casting.

### 2.2. Procedures

#### 2.2.1. Making of the Electrode and Membrane Preparation

The first stage in preparing the electrodes was to thoroughly polish the surface of the inner electrode, made of glassy carbon, which had a diameter of 3 mm. For this purpose, Al_2_O_3_ (with a particle diameter of 0.3 µm), wetted with deionized water, was used. The electrodes were then thoroughly rinsed with deionized water and placed in an ultrasonic bath to remove any residual Al_2_O_3_. The final degreasing of the surface was carried out by immersing the sensors in a THF solution.

**Electrodes without solid contact: ISE**

In order to optimize the qualitative composition, ten different membrane compositions were prepared and applied directly to the glassy carbon inner electrode. The qualitative and quantitative composition of the membrane mixtures was as follows: sensors No. 1–3, without ionophore, contained 3% *w*/*w* ion exchanger, 32% *w*/*w* PVC, and 65% *w*/*w* plasticizer; sensors No. 4–10 contained 2% *w*/*w* ion exchanger, 3% *w*/*w* ionophore, 65% *w*/*w* plasticizer, and 32% *w*/*w* PVC. These mixtures were dissolved in 2 mL of THF and then applied separately to the glassy carbon surface using a defined volume (30 µL) dropping method. The dripping process was carried out under controlled laboratory conditions, ensuring an equal volume of the applied mixture and consistent coverage of the sensor surface, whilst preventing air bubbles. The procedure was repeated three times, waiting approximately 20 min after each application. After coating the sensors with the membrane mixtures, they were left to dry at room temperature for 24 h, thereby allowing the solvent to evaporate and the membrane layer to stabilize.

**SP-ISE solid-contact electrodes**

To prepare the SP-ISE electrodes, multi-walled carbon nanotubes were introduced directly into the membrane mixture as one of the components. Five new mixtures (labeled 11–15) were prepared, the qualitative composition of which corresponded to ISE sensors No. 2, 3, 5, 8, 9. The qualitative composition of the membrane mixtures was as follows: sensors No. 11 and 12—3% *w*/*w* ion exchanger, 1% *w*/*w* MWCNTs, 31.5% *w*/*w* PVC, and 64.5% plasticizer; sensors No. 13–15—2% *w*/*w* ion exchanger, 1% *w*/*w* MWCNTs, 29.5% *w*/*w* PVC, 64.5% plasticizer, and 3% *w*/*w* ionophore. The membrane mixtures were mixed, homogenized, and applied in the same way as described in the preparation of the ISE electrodes. The dry membrane thickness of the SP-ISEs was approximately 0.30–0.26 mm, depending on the membrane composition.

**SC-ISE solid-contact electrodes**

The preparation of the SC-ISE electrodes consisted of applying a layer of carbon nanotubes directly to the electrode substrate as an intermediate layer prior to the application of the membrane. Before use, the MWCNT solution in THF at a concentration of 5 mg/ml was homogenized in an ultrasonic bath for approximately 10 min to achieve dispersion of the nanotubes and obtain a homogeneous system. The homogenized nanotube suspension was applied by drop-coating onto the ion-selective electrode surfaces, which had been previously polished and degreased (in THF). A volume of 10 µL of MWCNTs was applied to each sensor in a single drop. The electrodes were then left to dry at room temperature for 3 h to allow the solvent to evaporate and the nanotube layer to stabilize. Once the carbon nanotube layer had dried, the selected membrane mixtures were applied to the electrode surfaces as described in the section on ISE electrode preparation. The composition of the membrane mixtures was identical to that used for the ISE electrodes. The resulting electrodes were designated in the study as numbers 16–20.

All types of electrodes (ISE, SP-ISE, and SC-ISE) were conditioned in a 10^−3^ mol L^−1^ solution of the primary ion for three days after preparation and were subsequently stored in air at room temperature.

#### 2.2.2. Electrode Calibration

To determine the characteristics of the electrodes, a series of chlorhexidine standard solutions were prepared with the following concentrations: 10^−3^–10^−7^ mol L^−1^, as well as a solution with an average concentration of 5 × 10^−5^ mol L^−1^ for conditioning the electrodes prior to the measurements. The solutions for calibration of the electrodes were prepared in deionized water with a pH of 6.8. The tested sensors, together with a reference electrode, were immersed sequentially in solutions of increasing CHD concentrations. The measurements were carried out until the potential stabilized, stirring the solution using a magnetic stirrer.

#### 2.2.3. Determination of Chlorhexidine

Samples for determining chlorhexidine were prepared as follows:

Solutions of pure samples with a concentration of 1 × 10^−4^ mol L^−1^ were prepared by measuring predetermined volumes and diluting them in an acetate buffer solution with a pH of 4.5. Solutions of the Eludril samples (2.2 × 10^−4^ mol L^−1^) were prepared by transferring 10 mL of the Eludril solution into separate 100-mL volumetric flasks and making up to the mark with the acetate buffer solution at a pH of 4.5.

Standard solutions for determining chlorhexidine were prepared as follows:

To determine the calibration curve, solutions with concentrations of 10^−5^, 10^−4^, and 10^−3^ mol L^−1^ were prepared in the acetate buffer solution with a pH of 4.5. In the addition methods (the standard addition method and Gran’s method), the following solutions were used as standard solutions, respectively: a chlorhexidine solution with a concentration of 1 × 10^−3^ mol L^−1^ (for the determination of the pure compound) and a 2 × 10^−3^ mol L^−1^ standard solution for the determination of chlorhexidine in the preparation. Both standards were prepared in the acetate buffer solution with a pH of 4.5.

## 3. Results

### 3.1. The Effect of the Membrane Composition on the Response of the ISE Sensors

In order to determine the optimal membrane composition for the glassy carbon-based electrodes being developed, 10 different electrodes were constructed according to the procedure described in Section 2.2.1. Over the following days, calibration curves were determined; the calculated values of the linear regression coefficients are presented in Table 1.

All the electrodes exhibit near-Nernstian behavior in the CHD concentration range from 10^−5^ to 10^−6^ to 10^−3^ mol L^−1^. The average sensitivity ranges from 26 to 31 mV decade^−1^ with a standard deviation of 0.3–2.5 mV. The linear correlation coefficient ranges from 0.999 to 0.994. Based on the calibration curves obtained, it can be concluded that all the electrodes with an *o*-NPOE-plasticised membrane (with the exception of electrode No. 10) exhibit the widest linearity range and very good sensitivity of 29 mV decade^−1^. The electrodes containing DOS in the membrane exhibit sensitivity slightly lower than the linear range, at only 10^−5^–10^−3^ mol L^−1^. On the other hand, the electrodes with DDP, depending on the active substance, have a sensitivity of 26–31 mV decade^−1^ within a varying linear range.

In order to determine the kinetics of potential changes over time, the dynamic response was measured for specific electrodes.

**Figure 1:**
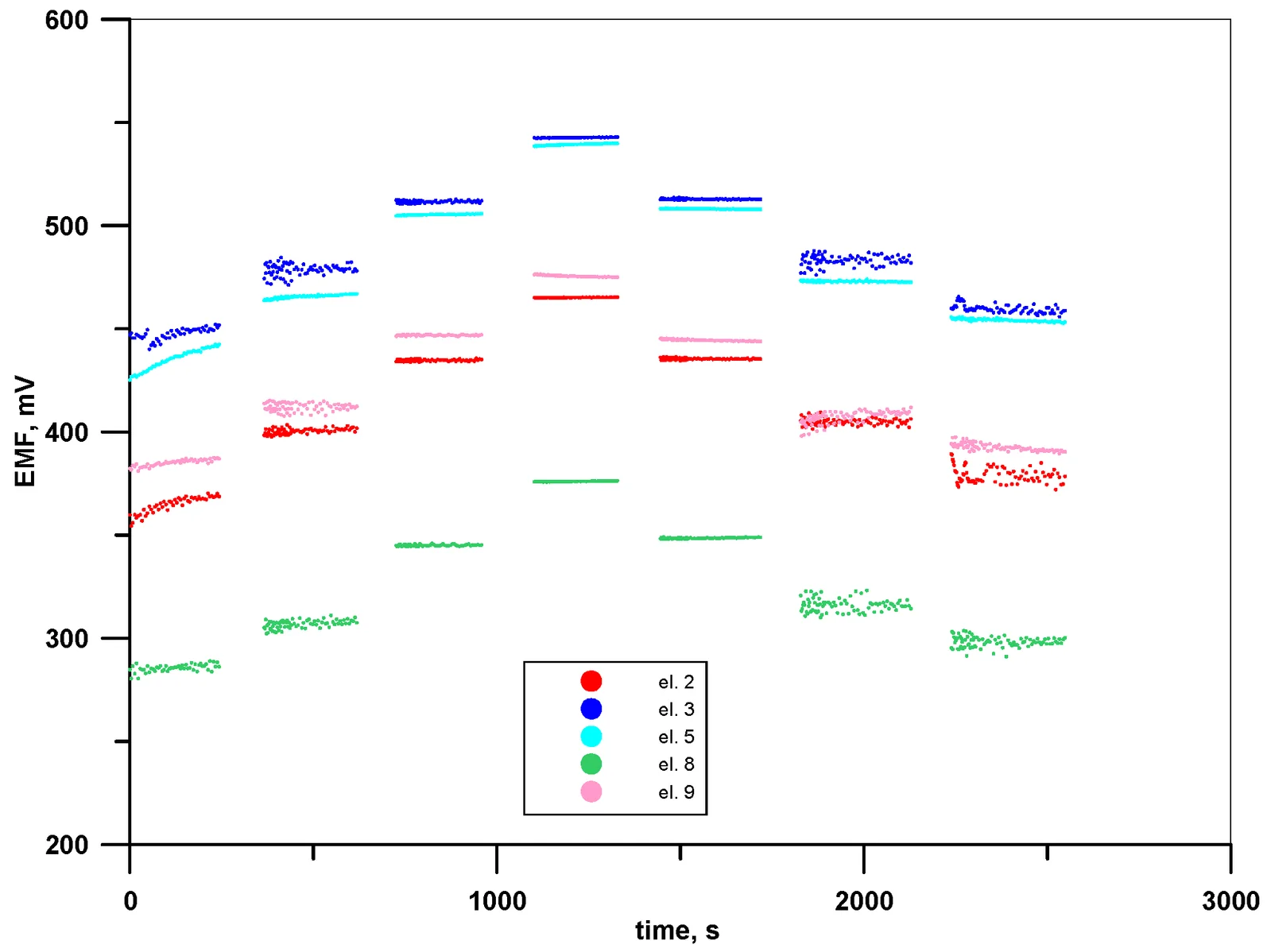
Dynamic response of electrodes 2, 3, 5, 8, and 9 in solutions with concentrations of 10^−3^ to 10^−6^ mol L^−1^.

The criteria for selecting the sensors were the type of ionophore and the widest linear range. Thus, electrode No. 5 with the DBC ionophore and electrodes Nos. 8 and 9 with the HSB*β*CD ionophore have a wide linear range: −log c = 6–3. For comparison, the so-called “blank” electrodes without ionophore (Nos. 2 and 3) were selected for further testing. Figure 1 shows the results for the selected optimal sensors 5, 8, 9, and 2 and 3. Based on the data presented, it can be concluded that the selected electrodes exhibit the most stable potential in the concentration range from 10^−4^ to 10^−3^ mol L^−1^. In lower concentration ranges, the potential is highly unstable, with a potential drift of several or even over a dozen mV being observed. It is associated with instability at the membrane/substrate interface and is consistent with the formation of an aqueous layer. Electrode 5 (DBC + *o*-NPOE) exhibits the most reproducible potential.

The electrodes’ response can be explained based on the physicochemical properties of plasticizers. Chlorhexidine ions, which have a charge that is highly dispersed over a large surface area and are inherently very lipophilic, may prefer plasticizers with a lower dielectric constant—such as diisodecyl phthalate—during the extraction process from the aqueous phase to the organic phase of the membrane. Additionally, an aromatic plasticizer will stabilize the organic ion being analyzed through π-interactions during this process. On the other hand, the high dielectric constant of 2-nitrophenyl octyl ether, ε = 24, facilitates the stabilization of charged ions in the membrane’s hydrophobic organic phase.

In the next part of the study, the primary aim was to evaluate whether the introduction of an intermediate layer could contribute to improving the analytical parameters of the electrodes, e.g., by enhancing their potential stability.

### 3.2. Characteristics of the SP-ISE and SC-ISE Sensors (Calibration Curves and Dynamic Response)

**Figure 2:**
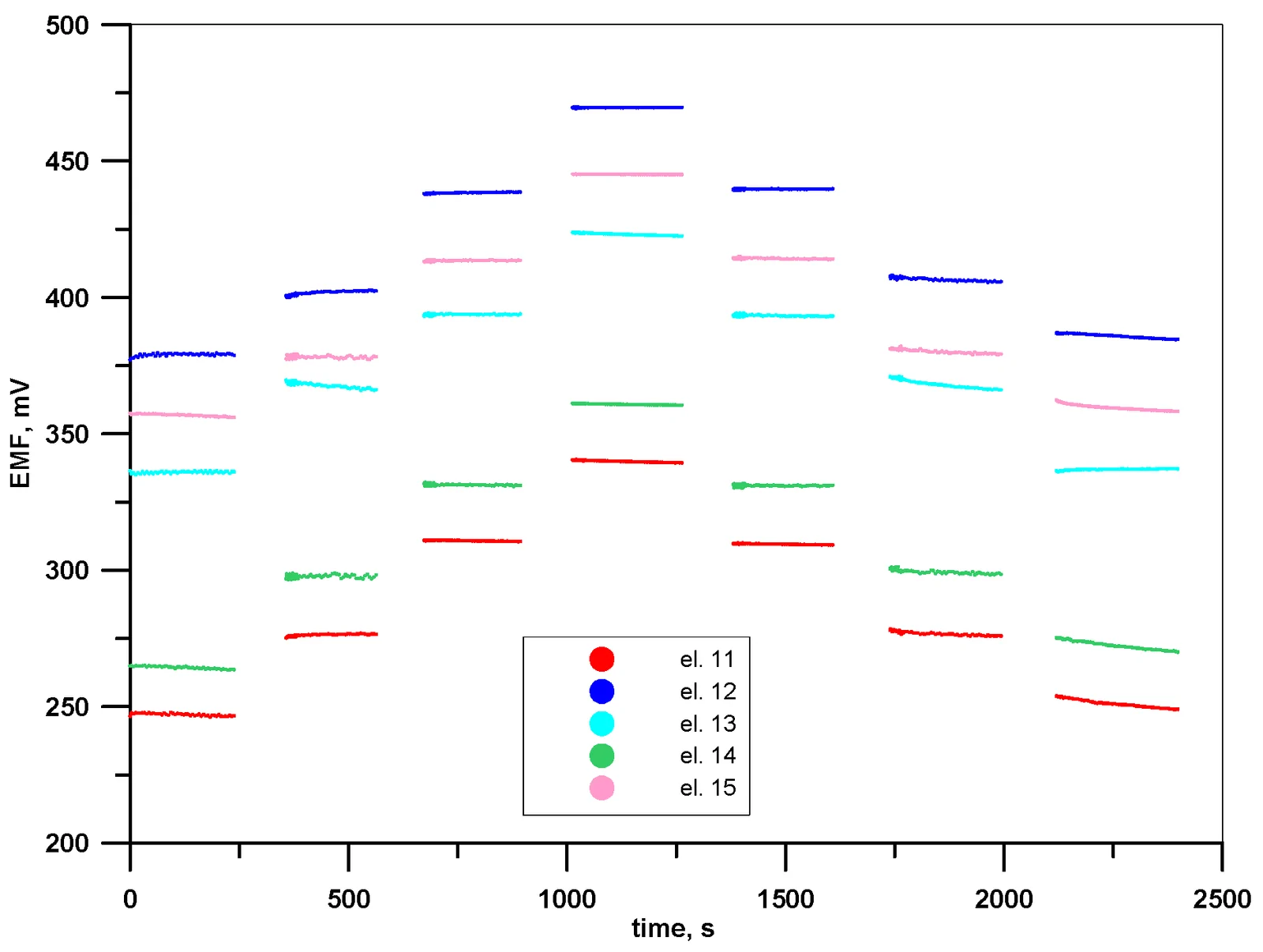
Dynamic response of electrodes no 11–15 in solutions with concentrations of 10^−3^ to 10^−6^ mol L^−1^.

Another group of electrodes consisted of the SP-ISE-type electrodes, in which a solid-contact layer was directly dispersed within the membrane. The procedure for preparing these sensors is described in Section 2.2.1. Electrodes No. 11–15, as shown in Table 2, exhibit very similar analytical parameters. The sensitivity of the electrodes ranges from 28 to 30 mV decade^−1^ with a standard deviation of 0.9–1.4 mV over a CHD concentration range of 10^−6^–10^−3^ mol L^−1^ for the electrodes containing HSB*β*CD in the membrane (electrodes No. 14 and 15), and for the other electrodes, 10^−5^–10^−3^ mol L^−1^. In this case, the addition of an ionophore acting as a “host” improves the linearity range. However, the calibration curves are not reproducible due to large shifts in standard potentials ranging from a dozen or so to as much as 40 mV. Figure 2 shows the dynamic response of the SP-ISE sensors. Compared with the previous series of the ISE electrodes, an improvement in potential stability can be seen, particularly at lower concentrations. The response to a change in concentration is quite good for all the electrodes and is similar. Although the incorporation of MWCNTs into the membrane improved potential stability at lower chlorhexidine concentrations, potential shifts were observed, which may adversely affect the reproducibility of the measurements. This trade-off was an important consideration in the subsequent development of the SC-ISE configuration with an intermediate solid-contact layer.

**Figure 3:**
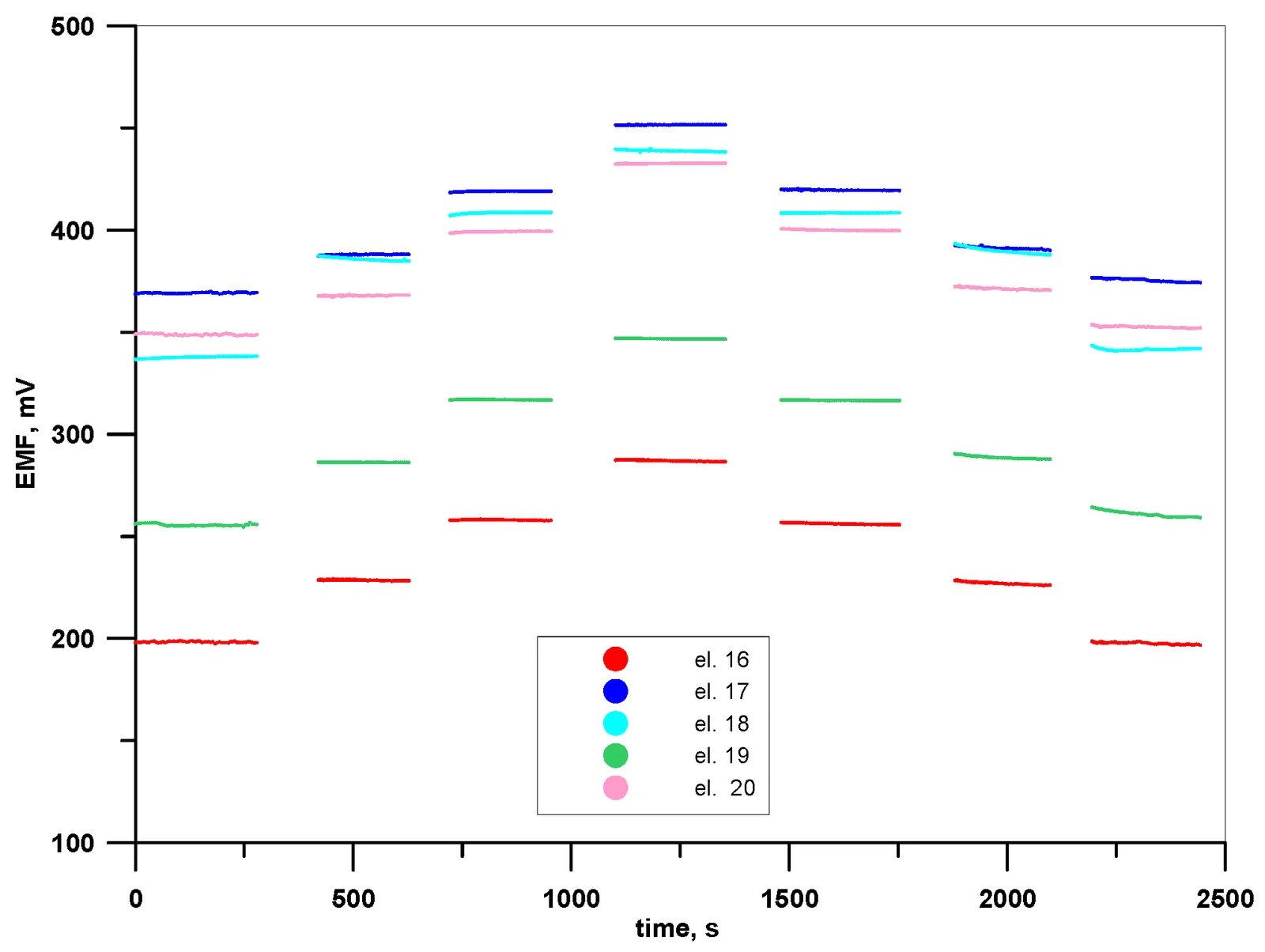
Dynamic response of electrodes no 16–20 in solutions with concentrations of 10^−3^ to 10^−6^ mol L^−1^.

The last group of electrodes consisted of the SC-ISE sensors (No. 16–20). All the electrodes exhibit a theoretical average slope of the calibration curve with a standard deviation of 0.6–1.7 mV in the CHD concentration range of 10^−6^–10^−3^ mol L^−1^ for the electrodes containing an ionophore, and 10^−5^–10^−3^ mol L^−1^ for the electrodes containing only an ion exchanger in the membrane. Calibrations of solid-contact electrodes generally exhibit a lower systematic error, which results in significantly lower standard deviations of their sensitivity compared to ISE electrodes. The standard potential of the electrodes exhibits a significantly lower standard deviation (within the range of approximately 4 to 9 mV), which is a major advantage compared to the SP-ISE electrodes. The detection limits achieved are relatively favorable for the three electrodes 18, 19, and 20 compared to the blank electrodes without SA (Table 3). The dynamic response curves of all the electrodes are similar, with electrode No. 20 exhibiting the most stable response (Figure 3). In summary, it can be concluded that all three electrodes (18–20) have very similar response curves relative to one another and exhibit parameters similar to those of the SP-ISE electrodes. The improved potential stability may be attributed to the MWCNT intermediate layer, which provides an electrically conductive interface between the glassy carbon substrate and the ion-selective membrane. This layer may reduce interfacial resistance and facilitate charge transfer, thereby contributing to the improved reproducibility and stability of the SC-ISE response.

### 3.3. Reversibility of the Electrodes

**Figure 4:**
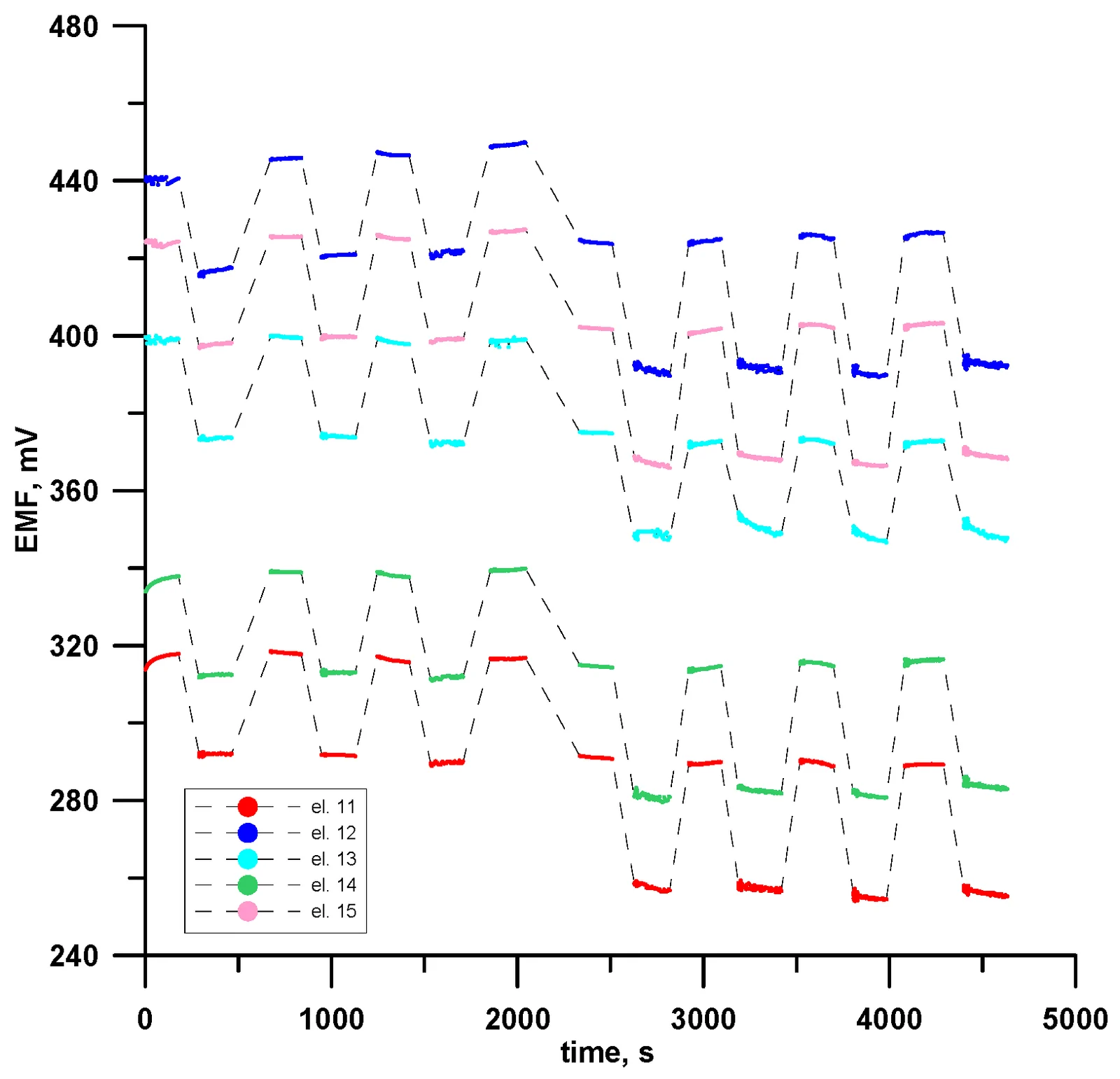
The reversibility test of the SP–ISE electrodes.

**Figure 5:**
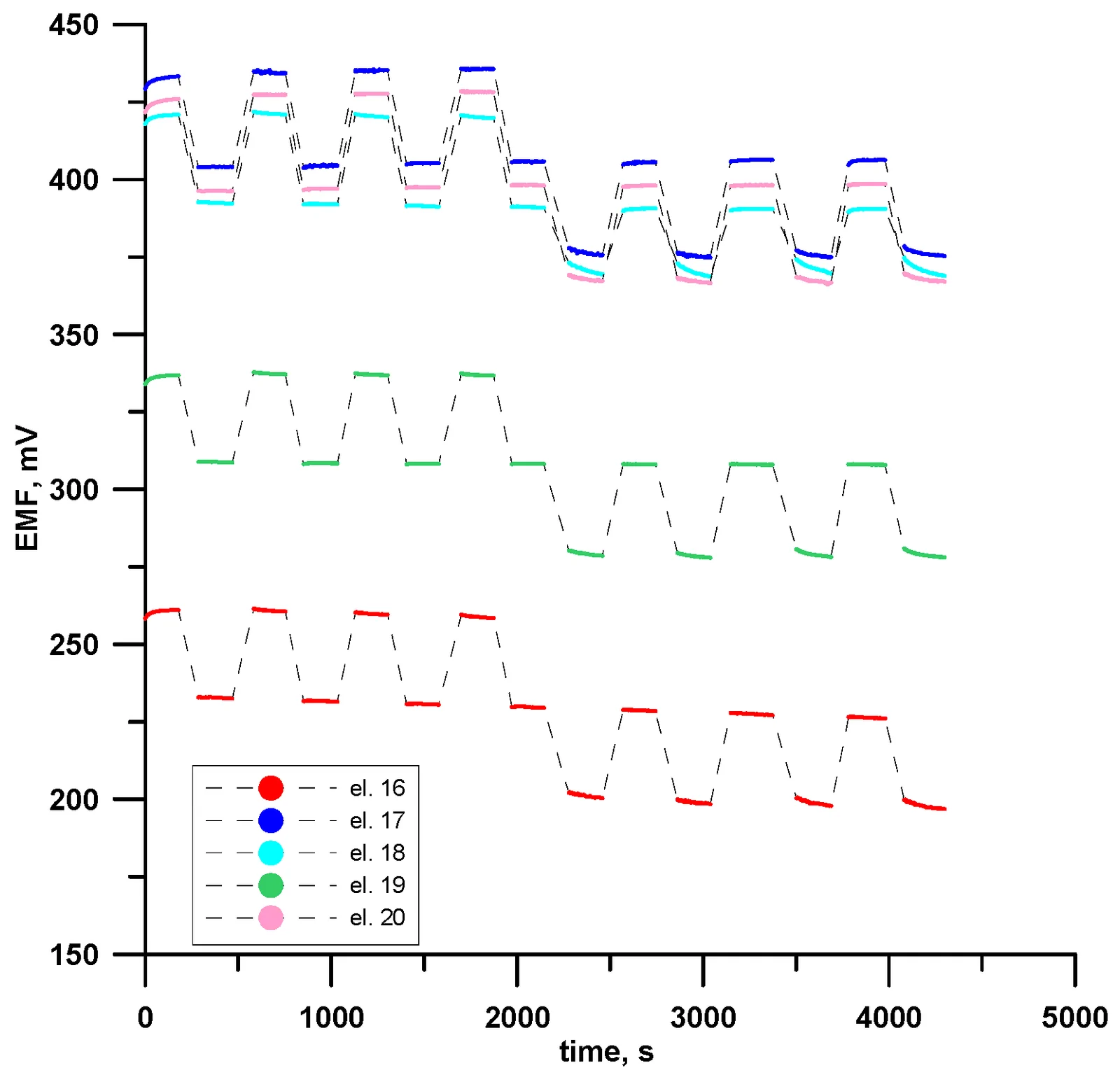
The reversibility test of the SC–ISE electrodes.

To investigate the reversibility of the potential, CHD solutions with concentrations of 10^−3^, 10^−4^, and 10^−5^ mol L^−1^ were used. The electrodes were immersed alternately in 10^−3^ and 10^−4^ mol L^−1^ solutions for a period of 3 min, and then in 10^−5^ mol L^−1^ and 10^−4^ mol L^−1^ solutions. The measurement data obtained are presented in Figure 4 and Figure 5 for the SP-ISE and SC-ISE electrodes, respectively.

For the SP-ISE electrodes, the potential generally shows high stability at a concentration of 10^−4^ mol L^−1^, particularly for electrodes 14 and 15. Electrode No. 15 (DDP + HSB*β*CD) exhibits a more stable potential compared with the blank electrode, without ionophore (No. 12). The mean potential for this electrode at the respective concentrations is E_1_ = 425.07; E_2_ = 399.68; E_3_ = 367.11 mV, with a standard deviation of approximately 1 mV. For electrode No. 12, the standard deviations of the mean potentials at the three specified CHD concentrations are approximately 1–3 mV. In turn, electrode 14 (*o*-NPOE + *β*CD) had an average potential with a standard deviation in solutions with concentrations of 10^−3^–10^−5^ mol L^−1^ as follows: E_1_ = 338.57 ± 1.12 mV; E_2_ = 312.96 ± 1.57 mV; and E_3_ = 281.63 ± 1.29 mV.

Figure 5 illustrates the reversibility of the potential over time for the electrodes with a solid-contact intermediate layer. When comparing the measurement results with those from the SP-ISE electrodes, better potential stability and a return to the initial value are evident, particularly for sensor 19, which has the same membrane composition as electrode 14 (*o*-NPOE + *β*CD). For this electrode, the calculated mean potential values at the same CHD solution concentrations as previously were as follows: E_1_ = 337.02 ± 0.53 mV; E_2_ = 308.46 ± 0.25 mV; and E_3_ = 279.17 ± 0.69 mV. Electrode 19 exhibited substantially lower standard deviations and better potential reversibility during measurements involving alternating chlorhexidine concentrations. These results further demonstrate the beneficial effect of the MWCNT intermediate layer on the stability and reversibility of the potentiometric response. It is also worth noting that electrodes 18 and 20 (with an ionophore in the membrane) also exhibit a very stable potential in a solution with an intermediate CHD concentration, with standard deviations of 0.54 and 0.71 mV, respectively, as well as in 10^−5^ mol L^−1^ 1.37 and 0.69 mV.

### 3.4. Response Time

**Figure 6:**
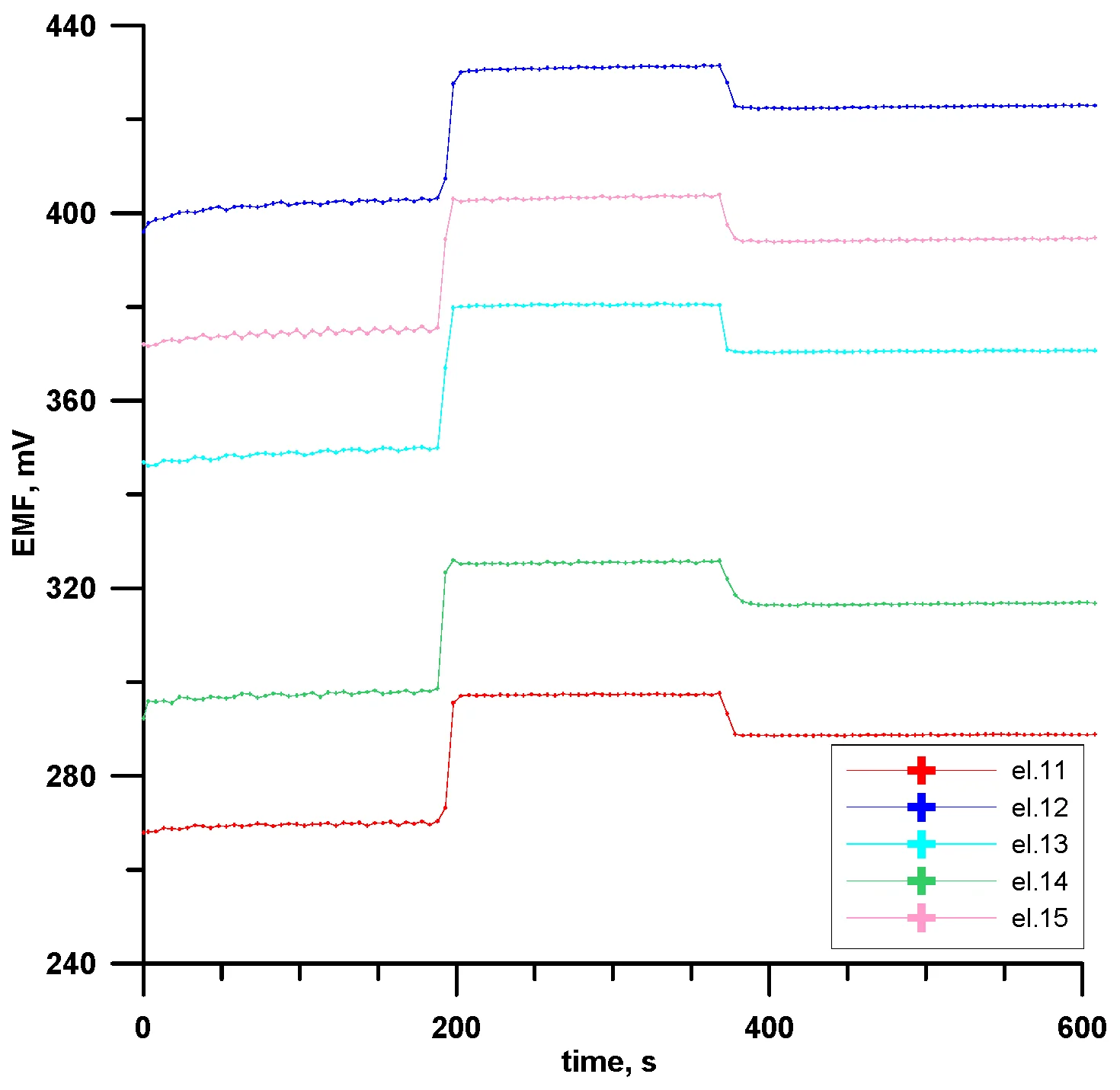
Response time of SP-ISE.

**Figure 7:**
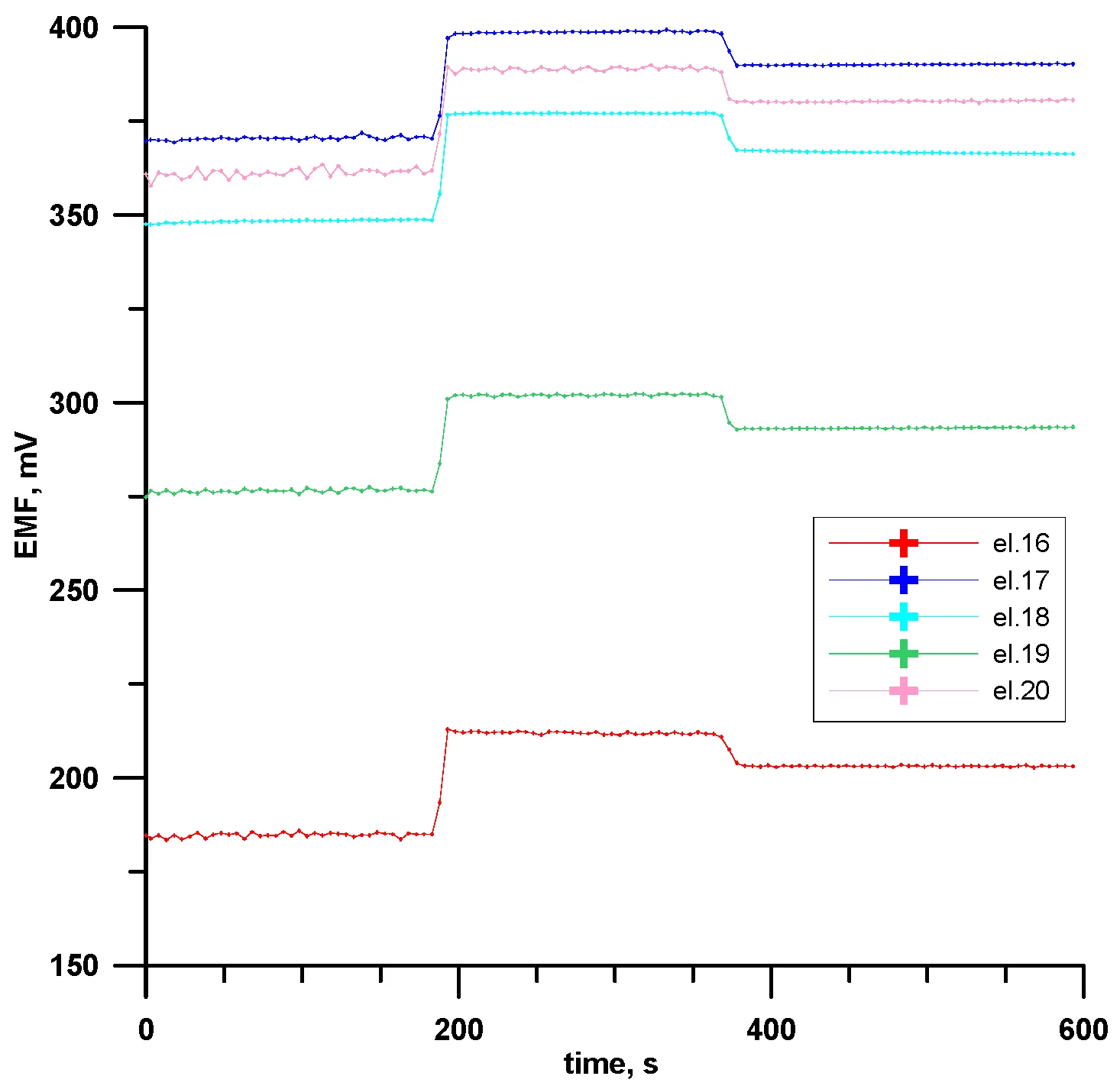
Response time of SC-ISE.

The response time was determined as a result of a sudden change in the concentration of the target ion in the solution. The electrodes were initially placed in a CHD solution with a concentration of 10^−5^ mol L^−1^ for approximately 3 min. The solution was stirred vigorously using a magnetic stirrer. Next, 1.5 mL of a chlorhexidine solution with a concentration of C = 10^−3^ mol L^−1^ was injected into the measuring vessel, and after 3 min, the solution was diluted in a 1:1 ratio. The recorded changes in potential as a function of time are shown in Figure 6 and Figure 7. The response time was estimated as the time required to achieve 95% of the final, stable potential value.

All the SC-ISE and SP ISE electrodes are characterized by a very short response time. During the concentration increase, it is approximately 10 s, and during dilution, 15 s. Furthermore, once a plateau is achieved, the signal is stable, and no significant potential drift is observed between changes in concentration.

### 3.5. Potential Drift

**Figure 8:**
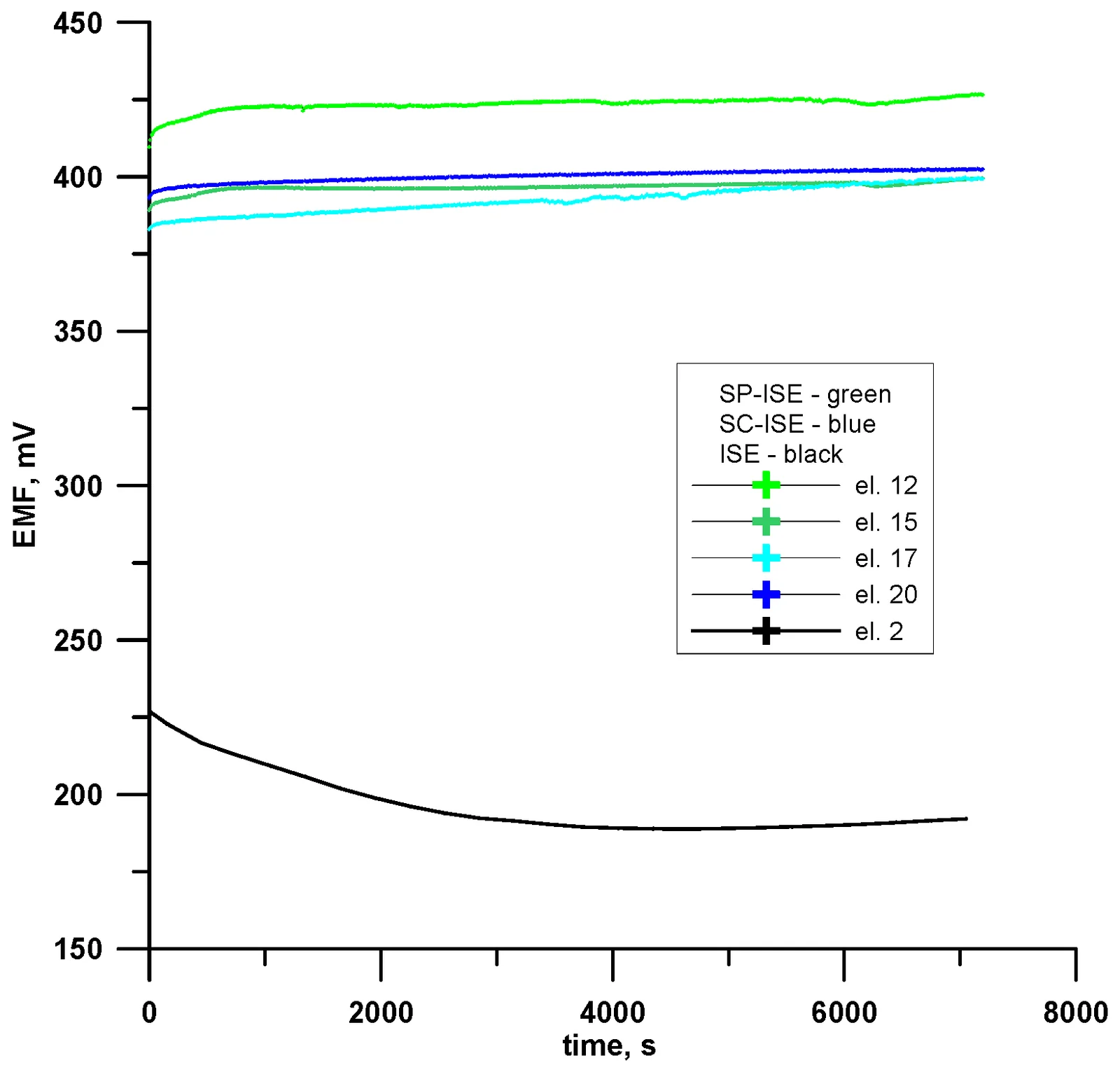
Drift in electrode potential over two hours.

**Figure 9:**
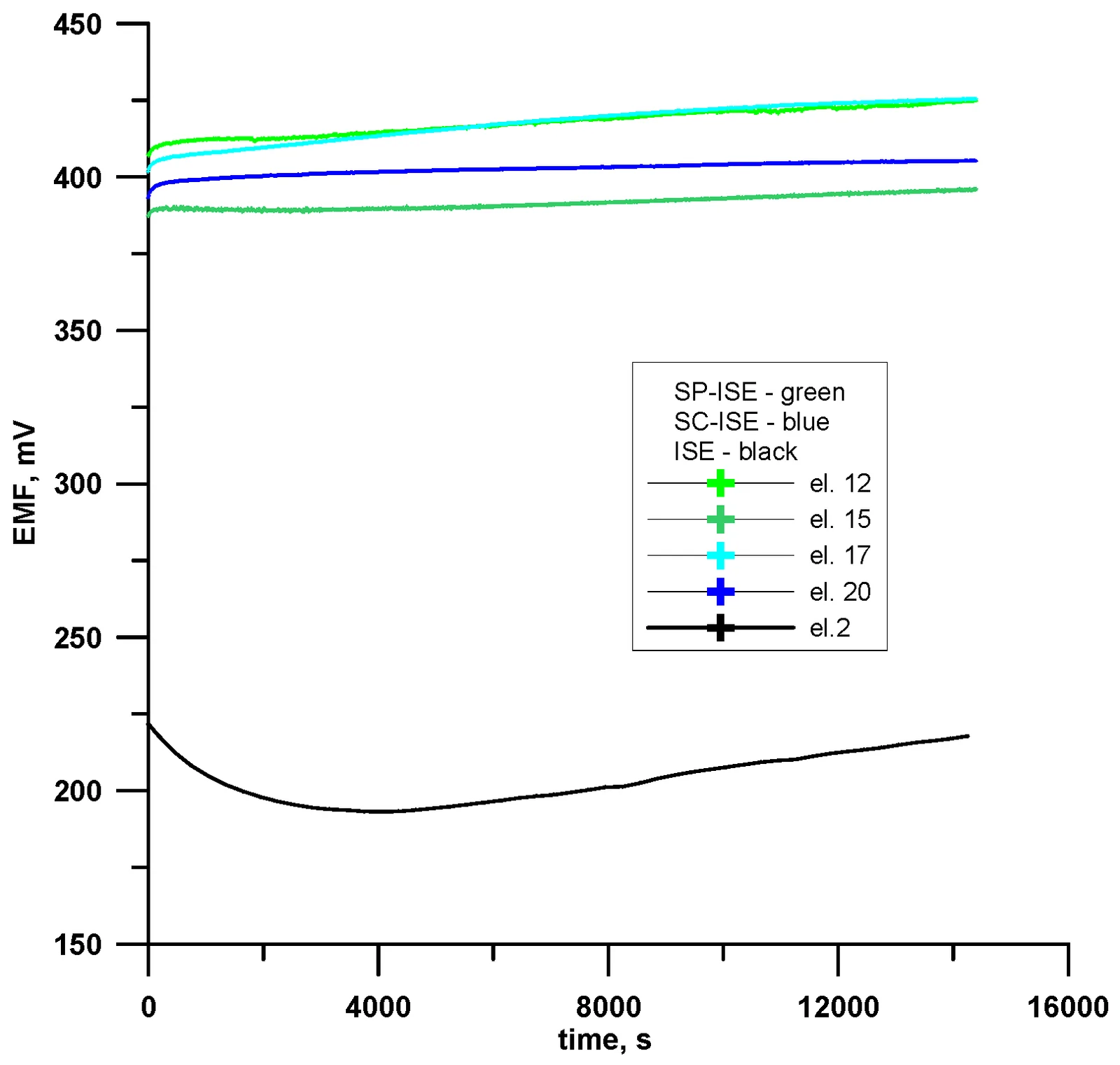
Drift in electrode potential over four hours.

Measurements of the long-term potential stability of the developed SP-ISE and SC-ISE electrodes were carried out. The sensors were immersed in a 10^−4^ mol L^−1^ CHD solution for 120 and 240 min (Figure 8 and Figure 9). The measurements were performed on two consecutive independent days. The potential drift was determined as the change in potential per unit time by analyzing the potential variation over the measurement period. In the membrane systems studied, the use of diisodecyl phthalate resulted in less potential drift than the use of NPOE, in both the SP-ISE and SC-ISE configurations. Therefore, the electrodes with a DDP-plasticized membrane are discussed in the following and next sections.

The SP-ISE electrode (No. 15) exhibited the best long-term stability. The potential drift over 120 min was 90 µV/min, whereas over 240 min it was 70 µV/min. For the remaining SP-ISE electrodes, the potential drift was up to 150 µV/min. The results obtained for the selected sensor (No. 15) and the sensor without ionophore (No. 12) are presented in Figure 6 and Figure 7, respectively. Similarly, among the SC-ISE electrodes, the electrode with the same qualitative membrane composition (No. 20) exhibited the best long-term stability. For this sensor, the potential drift over a 2-h period was 80 µV/min, whereas on the second measurement day, the drift over a 4 h period was 50 µV/min. The SC-ISE 20 exhibited excellent potential stability, with a low potential drift of approximately 50 µV min^−1^ during the 4 h measurement period. This low drift confirms the high stability of the developed electrode and supports its suitability for prolonged potentiometric measurements.

### 3.6. The Working pH Range

**Figure 10:**
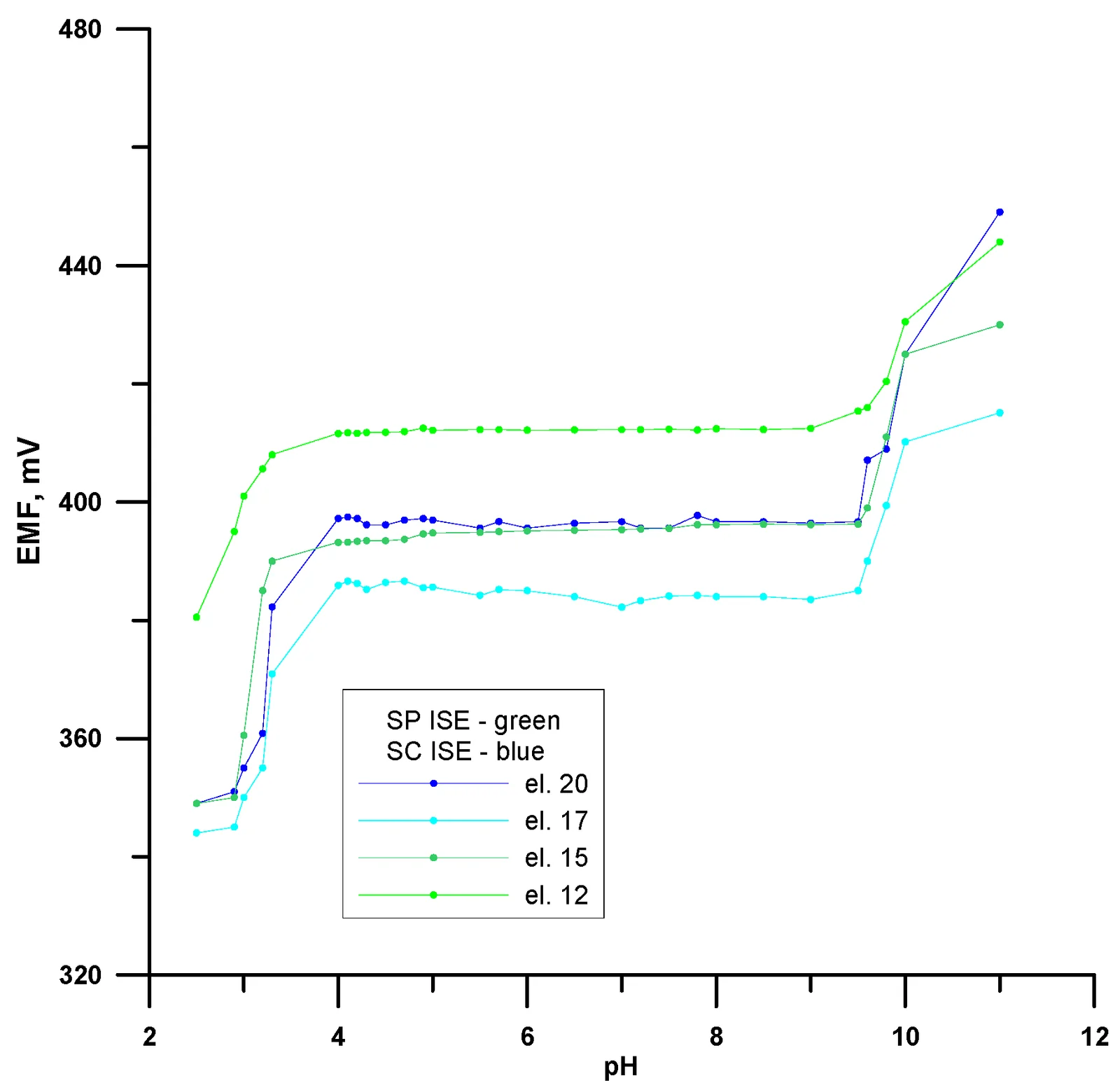
Dependence of the electrodes’ potential on pH.

The working pH range of the developed electrodes No. 20, 17, 15, and 12 was evaluated by gradually acidifying and alkalizing a 10^−4^ mol L^−1^ chlorhexidine solution. The pH of the solution was adjusted by successive additions of hydrochloric acid (HCl) and a base solution, while the electrode potential was continuously monitored. The obtained dependence of the electrode potential on pH is presented in Figure 10. As shown in this figure, the electrode potential remained relatively stable between approximately pH 4.0 and 9.5. Below pH 4, the decrease in potential may result from further protonation of chlorhexidine. At pH values above 9.5, the observed gradual increase in potential is likely related not only to the progressive deprotonation of chlorhexidine but also to the possible formation of the poorly soluble free-base form of chlorhexidine.

### 3.7. Selectivity

**Figure 11:**
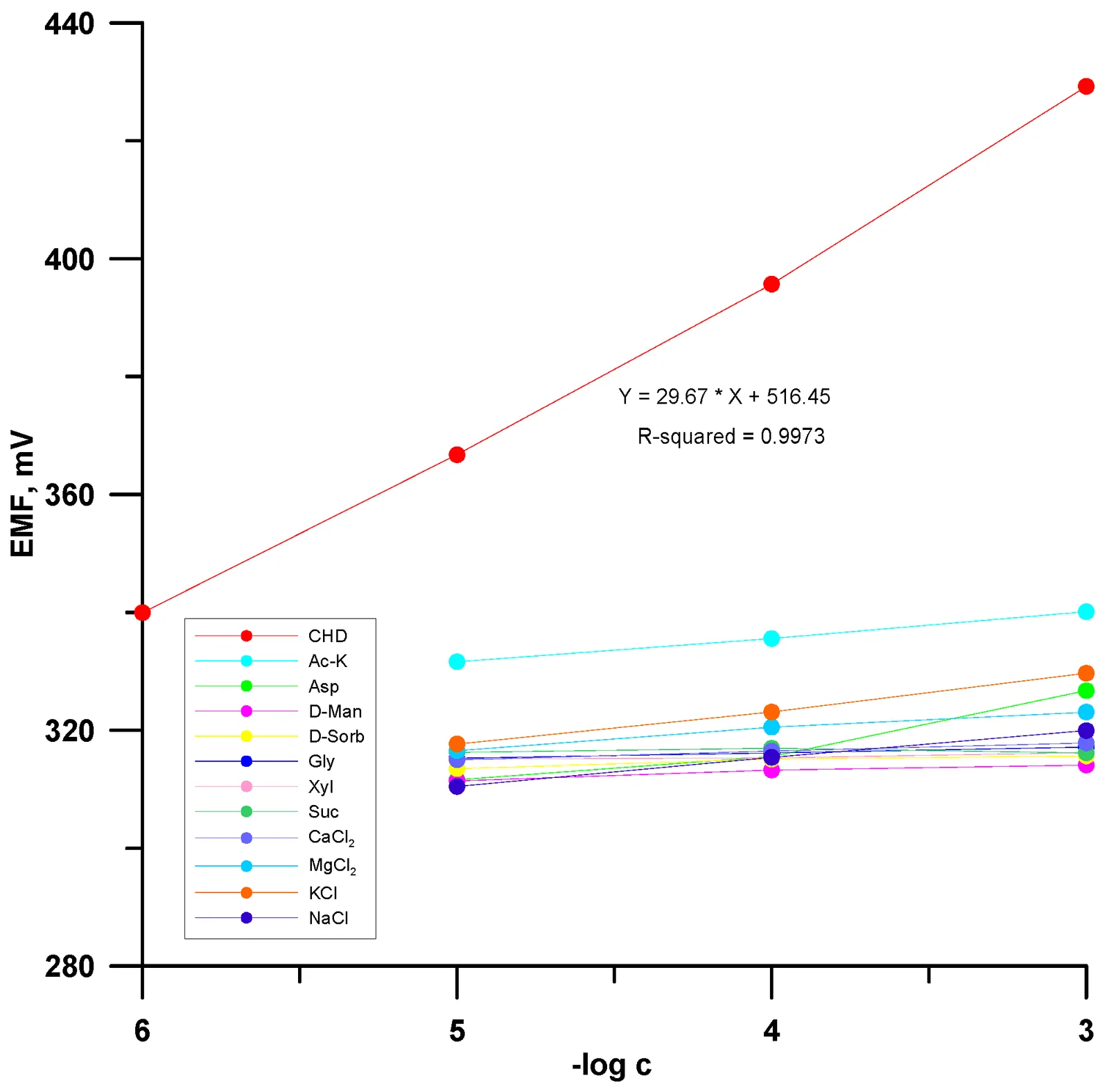
Selectivity of electrode No. 15 (SP-ISE).

**Figure 12:**
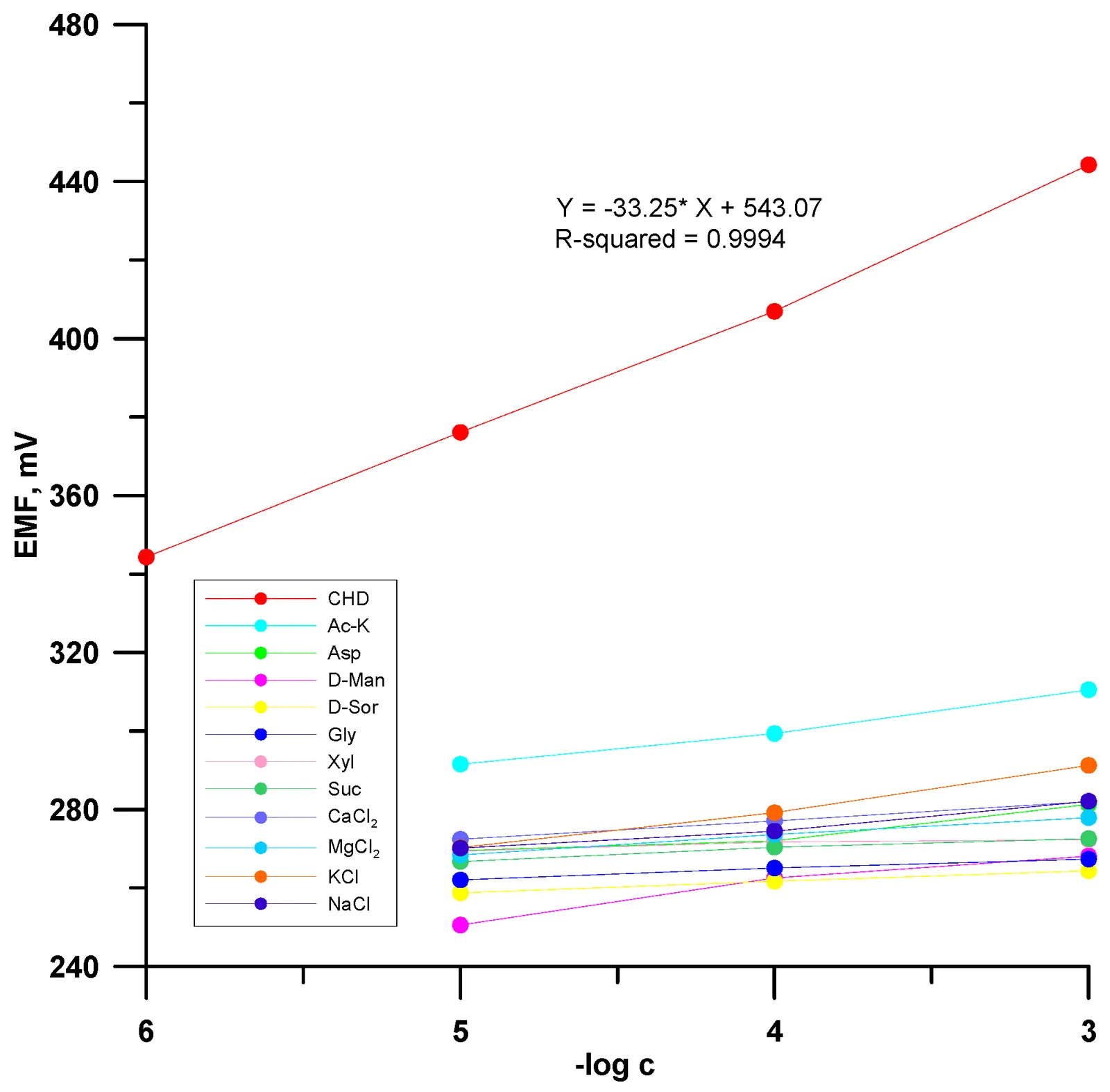
Selectivity of electrode No. 20 (SC-ISE).

The selectivity study was carried out using the separate solution method (SSM). To this aim, calibration curves were determined in solutions containing the main ion, interfering substances, such as acesulfame, D-mannitol, D-sorbitol, sucralose, propylene glycol, xylitol, and aspartame, and inorganic ions, including magnesium, calcium, sodium, and potassium. The selectivity of the electrodes was assessed based on the potential changes as a function of interferent concentration and by comparing the obtained characteristics with the electrode response to the CHD ion. The results of the potential–ion concentration relationships obtained for the two selected sensors, SP-ISE No. 15 and SC-ISE No. 20, are presented in Figure 11 and Figure 12. For the inorganic ions Na^+^, K^+^, Ca^2+^, and Mg^2+^, the selectivity coefficients were calculated using Bakker’s method [45] (Table 4). This method is a type of separate solution method as it involves determining calibration curves in a solution of the target ion and solutions of interfering ions. This method consists of extrapolating the response functions to c_I_ = c_J_ = 1 mol L^−1^ and application of the following equation:

$$\log K_{i/j}^{\mathrm{pot}}=\frac{E_{j}^{0}-E_{i}^{0}}{S}$$

where Ej^0^ is the potential of the electrode in the interfering ion’s solution; E_i_^0^ is the potential of the electrode in the main ion’s solution; S is the main ion’s slope of characteristics; z_i_ is the charge of the main ion; and z_j_ is the charge of the interfering ions.

Using this method, the charge of the target ions and interferents is omitted from the Nikolsky–Eisenman equation when calculating the selectivity coefficient.

All the sensors studied showed good selectivity toward the investigated interfering ions. Potassium ions originating from KCl and potassium acesulfame exhibited the greatest influence on the electrode potential, followed by sodium ions and the divalent cations Mg^2+^ and Ca^2+^.

The highest values of the selectivity coefficients obtained by the separate solution method according to Bakker et al. were $K_{\mathrm{CHD}/\mathrm{Na}}^{\mathrm{pot}}=10^{-6}$, KCHD/Kpot=10−5 KCHD/Capot=10−7 and $K_{\mathrm{CHD}/\mathrm{Mg}}^{\mathrm{pot}}=10^{-7}$. These values indicate a strong preference of the electrode for CHX over the investigated inorganic cations. Considering that typical concentrations of Na^+^, K^+^, Ca^2+^, and Mg^2+^ in saliva are in the millimolar range [46], substantially lower than the reference concentration used for the determination of the selectivity coefficients, no significant interference from these ions is expected under physiologically relevant conditions.

The investigated organic compounds, including sweeteners and polyhydric alcohols, produced a substantially weaker potentiometric response than CHX over the concentration range of 10^−5^–10^−3^ mol L^−1^, as indicated by the comparative calibration curves.

These results support the suitability of the developed sensor for the analysis of chlorhexidine-containing pharmaceutical formulations and its potential application to real pharmaceutical samples.

### 3.8. Water Layer Test

The undesirable accumulation of water at the interface between the solid-contact layer and the ion-selective membrane may negatively affect the potentiometric response of an electrode. Such a water layer may originate from residual moisture in the solvent used during membrane preparation or from water diffusing from the aqueous sample through the ion-selective membrane [47]. The composition of the interfacial aqueous phase may vary depending on the composition of the solution in contact with the membrane. Both primary and interfering ions can migrate through the membrane and accumulate at the solid-contact/membrane interface. The formation of such an aqueous layer can lead to potential instability and affect the long-term performance of solid-contact ion-selective electrodes. Therefore, the water layer test is commonly employed to assess whether an aqueous phase has formed between the ion-selective membrane and the solid contact.

**Figure 13:**
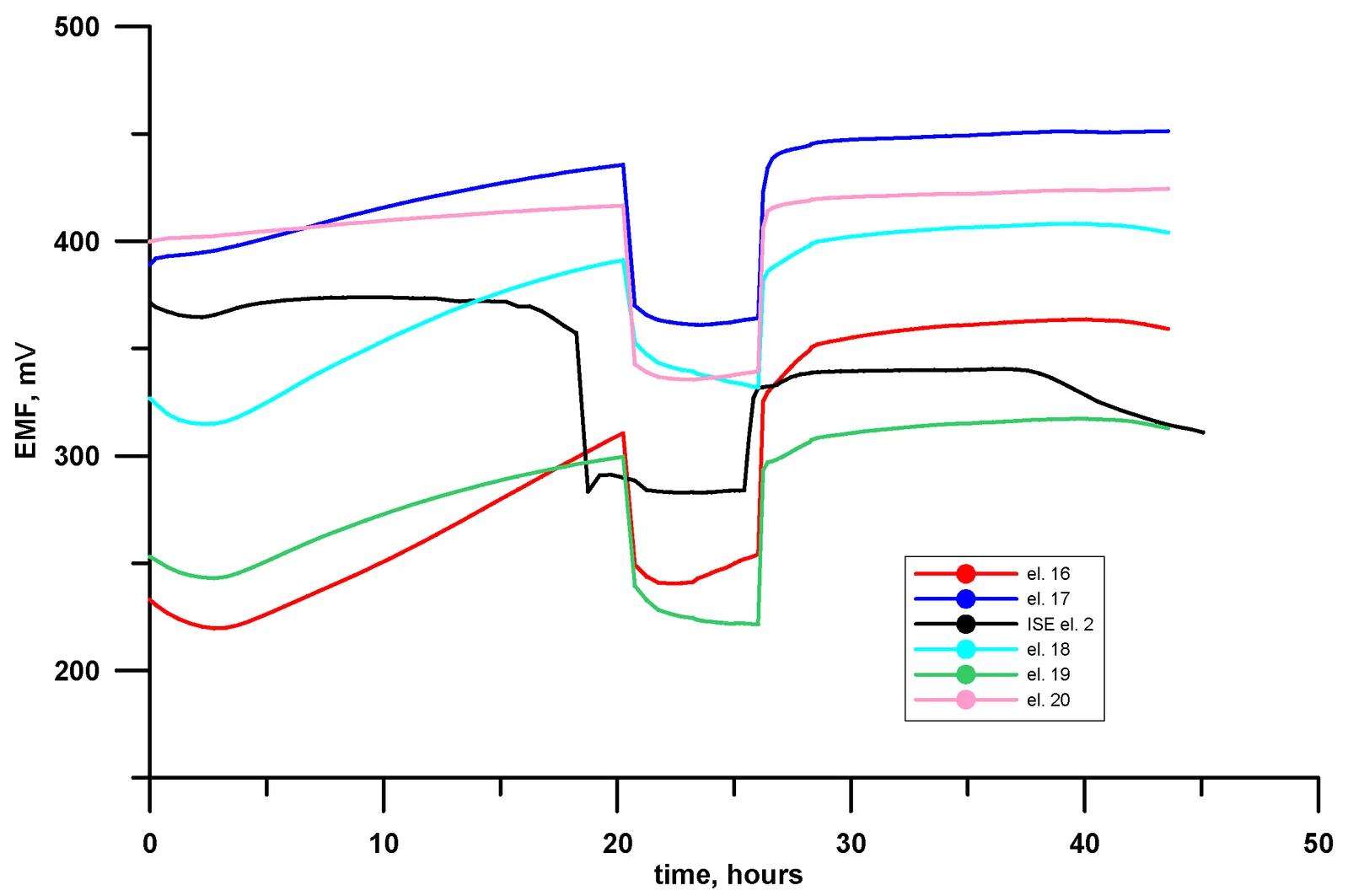
The water layer test of the SC-ISEs with control (ISE).

The water layer test was performed to investigate whether a thin aqueous layer had formed at the interface between the ion-selective membrane and the solid-contact layer. The test was conducted using a three-step solution-change procedure. In the initial stage, the electrodes were immersed in a 10^−3^ mol L^−1^ chlorhexidine solution for 22 h, while the potential response was continuously recorded. Subsequently, the solution was replaced with a 10^−3^ mol L^−1^ KCl solution containing the most strongly interfering ion, and the measurements were continued for an additional 5 h. After this period, the solution was replaced again with the primary ion solution, and the potential was monitored for a further 22 h to assess the recovery of the potential toward its initial value and the stability of the potentiometric response. The potential changes recorded over approximately 50 h are presented in Figure 13.

The electrodes incorporating DDP-plasticized membranes exhibited the most stable potential response. Among these electrodes, electrode No. 20, containing the lipophilic ionophore cyclodextrin, showed the most favorable characteristics. The increased lipophilicity of the membrane, combined with the carbon nanomaterial intermediate layer, may contribute to a more hydrophobic electrode interface and effectively suppress the formation of an aqueous layer. These results highlight the beneficial effect of the MWCNT intermediate layer and membrane composition on the stability of the membrane/solid-contact interface. In contrast, electrodes No. 16, 18, and 19 exhibited a slight potential drift during exposure to the interfering-ion solution and upon returning to the chlorhexidine solution. These changes may indicate the formation of an undesirable aqueous layer at the membrane/solid-contact interface.

### 3.9. Lifetime

The lifetime was evaluated by measuring the slopes of the calibration characteristics of the electrodes stored in air at room temperature. Measurements were generally performed every 7–14 days in a chlorhexidine solution (Table 5). The studies of the SP-ISE electrodes showed that all the sensors retained near-theoretical analytical parameters after 7 months. The SC-ISE electrodes also maintained stable calibration characteristics over the same period, demonstrating the long-term durability of both electrode configurations. Compared with the SC-ISE series, the SP-ISE electrodes exhibited slightly greater variations in their analytical parameters over time; however, they still maintained a good potentiometric response. These results indicate that the actual lifetime of the investigated sensors is likely to exceed the period covered by the present study. Therefore, further monitoring of the stability of the analytical parameters of the electrodes is planned over the subsequent weeks of use.

Both the SP-ISE and SC-ISE configurations maintained stable calibration characteristics during prolonged storage in dry air. These results further support the practical applicability of the developed sensors for long-term potentiometric measurements.

### 3.10. Summary of the Obtained Good Sensors and Analytical Application

To summarize the results obtained in this study, 10 types of SP-ISE and SC-ISE electrodes were produced, which differ in certain analytical parameters. Since there was no single electrode that performed perfectly—i.e., had the best parameters—specific criteria were used to select candidate sensors. Although other sensors, such as No. 14 and 18, also achieved good parameters such as reversibility, the goal was to identify a single membrane composition—an electrode that exhibits mostly good parameters defining its potential (stability, reversibility, the highest possible precision, and a low standard deviation)—and to compare this composition in two designs. Table 6 below presents a comparison of the selected electrodes.

The selected SP-ISE and SC-ISE sensors have the widest linearity range. The SC-ISE sensors show a much smaller standard deviation from E^0^ (sensors 18 and 19 are highlighted). From a comparison of Figure 2 and Figure 3, it can be concluded that at concentrations of 10^−6^, 10^−5^, 10^−4^, and 10^−3^, electrodes 15 and 20 exhibited the most stable and reproducible potentiometric response. In terms of potential reversibility, the SC-ISE electrodes exhibited a very low standard deviation (sensor 18) compared to the SP-ISE electrodes (>1 mV). The water layer test effectively determined the selection of further sensors, as among all the SC-ISE electrodes, sensor 20 exhibited the most optimal potential response in a solution containing both the main and interfering ions, indicating a limited presence of a water layer. Further studies of potential drift, pH dependence, and selectivity focused on sensor 20 and the corresponding sensor No. 15, which had the same membrane composition. For comparison and to determine whether the ionophore influenced specific parameters, blank electrodes were also selected for the study. Based on the results of the study, it can be concluded that the selected parameters are relatively optimal for electrode No. 20.

To assess the analytical applicability of sensor No. 20 for the determination of chlorhexidine in real and pharmaceutical matrices, its performance was evaluated using pure chlorhexidine solutions and the commercial pharmaceutical formulation Eludril. Quantitative analyses were performed using the calibration curve, standard addition, and Gran’s methods. The results, summarized in Table 7, were consistent with the analytical performance typically associated with ion-selective electrode-based methods. Based on three replicate determinations, the chlorhexidine recoveries from the pharmaceutical formulation ranged from 95.9 to 98.6%, while the precision, expressed as the standard deviation, ranged from 1.4 to 7.3 mg L^−1^ for the standard addition method. The consistency of the results obtained using the three complementary quantitative approaches further supports the analytical applicability of sensor No. 20 for the determination of chlorhexidine in the investigated pharmaceutical formulation.

### 3.11. Comparison Between the Proposed Ion-Selective Electrode and the Reported Ones

As reported in the literature, several potentiometric sensors have been developed for the determination of chlorhexidine using different electrode designs. Although the analytical characteristics of the previously reported sensors are generally comparable to those of the proposed electrode, the main novelty of the present work lies in the electrode architecture, particularly in the incorporation of an MWCNT-based transducing layer onto a glassy carbon substrate.

The MWCNT intermediate layer may contribute to improved potentiometric performance of the SC-ISE by providing an electrically conductive interface between the glassy carbon substrate and the ion-selective membrane. Its incorporation was associated with improved potential stability and reversibility compared with the other investigated electrode configurations.

An important advantage of the proposed SC-ISE is its low potential drift, indicating high potential stability and limited susceptibility to the formation of an aqueous layer. The combination of the MWCNT solid-contact layer with the optimized membrane composition therefore represents the main engineering contribution of the present design. A comparison of the analytical characteristics and the performance of the proposed and previously reported chlorhexidine sensors is presented in Table 8.

## 4. Conclusions

In this study, an innovative all-solid-state ion-selective electrode was developed based on a glassy carbon electrode with an intermediate layer of multi-walled carbon nanotubes (GCE/MWCNT/HSBβCD) and a PVC-based ion-selective membrane for the potentiometric determination of chlorhexidine. Among the investigated sensors, SC-ISE No. 20 exhibited the most favorable overall performance, including a stable potentiometric response, good selectivity toward chlorhexidine, and limited susceptibility to the formation of an aqueous layer at the membrane/solid-contact interface. The electrode also demonstrated good long-term stability and potential reversibility. The favorable selectivity and stability characteristics enabled the electrode to be successfully applied to the determination of chlorhexidine in the commercial pharmaceutical formulation Eludril. The consistent results obtained using the calibration curve, standard addition, and Gran’s methods further support the practical applicability of SC-ISE No. 20 for the analysis of real pharmaceutical samples. These results indicate that the GCE/MWCNT architecture represents a promising solid-contact design for potentiometric sensors selective toward chlorhexidine and potentially other pharmaceutical compounds.

## Figures and Tables

**Table 1:** Parameters of the linear regression curves for the ISE electrodes. LR—the linear range of the calibration plot (mean ± SD, the number of repeated calibrations for a particular electrode n = 3).

El. No.	Kind of Ionophore	Plasticizer	Lipophilic Additive	a	b	LR	R^2^	LOD
1	-	DOS	KTpClBP	29.59 ± 1.29	634.24 ± 5.32	10^−5^–10^−3^	0.9981 ± 0.0031	6.5 × 10^−4^ ± 2.3
2	-	*o*-NPOE	KTpClBP	29.34 ± 2.33	551.18 ± 8.89	10^−6^–10^−3^	0.9993 ± 0.0005	8.9 × 10^−6^ ± 3.6
3	-	DDP	KTpClBP	28.79 ± 2.56	549.14 ± 9.85	10^−5^–10^−3^	0.9992 ± 0.0007	9.8 × 10^−7^ ± 1.4
4	DBC	DOS	KTpClBP	27.67 ± 1.87	535.50 ± 9.31	10^−5^–10^−3^	0.9992 ± 0.0011	6.2 × 10^−6^ ± 2.8
5	DBC	*o*-NPOE	KTpClBP	29.07 ± 0.85	642.51 ± 4.43	10^−6^–10^−3^	0.9986 ± 0.0006	8.3 × 10^−7^ ± 1.6
6	DBC	DDP	KTpClBP	29.97 ± 2.28	529.41 ± 12.51	10^−5^–10^−3^	0.9943 ± 0.0050	7.8 × 10^−4^ ± 2.5
7	HSB*β*CD	DOS	KTpClBP	27.76 ± 0.93	547.44 ± 8.31	10^−5^–10^−3^	0.9970 ± 0.0012	5.6 × 10^−4^ ± 1.2
8	HSB*β*CD	*o*-NPOE	KTpClBP	28.66 ± 1.19	593.78 ± 8.62	10^−6^–10^−3^	0.9986 ± 0.0006	8.6 × 10^−7^ ± 0.2
9	HSB*β*CD	DDP	KTpClBP	26.11 ± 0.27	612.82 ± 1.06	10^−6^–10^−3^	0.9971 ± 0.0005	1.6 × 10^−6^ ± 1.2
10	ETH 117	*o*-NPOE	KTpClBP	30.18 ± 0.79	620.86 ± 7.14	10^−5^–10^−3^	0.9976 ± 0.0023	4.5 × 10^−4^ ± 3.1

**Table 2:** Parameters of the linear regression curves for the SP-ISE electrodes (data obtained within 2 weeks of the electrodes being prepared). LOD—limit of detection, mol L^−1^, R—reproducibility, mean for two sensors (slope ± SD, mV decade^−1^).

El. No.	Ionophore	Plasticizer	n	a	b	LR	R^2^	LOD	R
11	-	*o*-NPOE	6	30.94 ± 1.01	446.38 ± 41.73	10^−5^–10^−3^	0.9989 ± 0.0011	1.0 × 10^−6^ ± 2.4	29.15 ± 1.30
12	-	DDP	6	28.94 ± 0.95	544.75 ± 19.46	10^−5^–10^−3^	0.9993 ± 0.0007	1.2 × 10^−6^ ± 0.6	27.68 ± 1.15
13	DBC	*o*-NPOE	6	28.38 ± 1.58	510.64 ± 32.40	10^−5^–10^−3^	0.9984 ± 0.0017	6.3 × 10^−7^ ± 1.6	28.10 ± 1.70
14	HSB*β*CD	*o*-NPOE	6	29.72 ± 0.95	472.17 ± 35.12	10^−6^–10^−3^	0.9989 ± 0.0011	6.3 × 10^−7^ ± 0.3	29.15 ± 1.12
15	HSB*β*CD	DDP	6	28.72 ± 1.40	534.94 ± 17.70	10^−6^–10^−3^	0.9989 ± 0.0005	7.9 × 10^−7^ ± 0.8	28.35 ± 1.69

**Table 3:** Parameters of the linear regression curves for the SC-ISE electrodes (data obtained within 2 weeks of the electrodes being prepared). SA—active substance; LA—lipophilic additive; R—reproducibility, mean for two sensors (slope ± SD, mV decade^−1^).

El. No.	SA	Plasticizer	LA	n	a	b	LR	R^2^	LOD	R
16	-	*o*-NPOE	KTpClBP	5	29.18 ± 0.82	473.41 ± 4.55	10^−5^–10^−3^	0.9992 ± 0.0021	1.2 × 10^−6^ ± 0.3	29.06 ± 0.92
17	-	DDP	KTpClBP	5	30.53 ± 1.06	552.04 ± 5.30	10^−5^–10^−3^	0.9991 ± 0.0009	1.6 × 10^−6^ ± 0.5	28.78 ± 1.20
18	DBC	*o*-NPOE	KTpClBP	4	29.61 ± 0.59	543.09 ± 7.16	10^−6^–10^−3^	0.9996 ± 0.0004	4.0 × 10^−7^ ± 1.0	29.14 ± 0.71
19	HSB*β*CD	*o*-NPOE	KTpClBP	4	29.53 ± 0.86	478.41 ± 4.36	10^−6^–10^−3^	0.9991 ± 0.0009	7.9 × 10^−7^ ± 0.8	29.43 ± 0.41
20	HSB*β*CD	DDP	KTpClBP	5	29.44 ± 1.75	539.36 ± 9.04	10^−6^– 10^−3^	0.9973 ± 0.0027	6.3 × 10^−7^ ± 0.9	29.31 ± 1.29

**Table 4:** Results for selectivity coefficients (c_i_ = c_j_, 1 mol L^−1^).

Electrode No.	Selectivity Coefficient $K_{\mathrm{CHD}/j}^{\mathrm{pot}}$				
	Acesulfame-K	KCl	NaCl	CaCl_2_	MgCl_2_
12 SP-ISE	2.9 × 10^−6^	3.8 × 10^−6^	1.6 × 10^−6^	7.0 × 10^−7^	9.0 × 10^−7^
15 SP-ISE	3.0 × 10^−6^	2.0 × 10^−6^	7.2 × 10^−7^	2.83 × 10^−7^	6.6 × 10^−7^
17 SC-ISE	1.9 × 10^−5^	1.6 × 10^−6^	2.5 × 10^−7^	1.1 × 10^−7^	1.2 × 10^−7^
20 SC-ISE	7.1 × 10^−7^	2.3 × 10^−7^	4.7 × 10^−8^	3.8 × 10^−8^	2.9 × 10^−8^

**Table 5:** Change in the slope characteristics of the SP-ISE and SC-ISE electrodes over time.

El. No.	11	12	13	14	15	16	17	18	19	20
Time, Weeks	Slope of Characteristic, mV Decade^−1^									
6	25.4	29.7	26.5	25.9	29.9	26.5	29.9	24.4	29.0	28.7
7	25.2	26.8	26.2	26.8	26.7	25.8	26.7	30.9	25.4	26.7
8	27.4	30.0	28.9	25.9	27.9	27.8	30.3	29.1	28.0	28.9
10	26.5	28.5	27.4	27.2	28.7	26.0	30.3	27.2	26.8	28.5
12	25.2	26.8	26.0	38.8	26.6	25.9	26.8	32.2	25.4	26.7
14	25.6	26.8	26.5	25.5	26.5	28.5	29.5	32.4	29.5	29.5
17	25.5	27.5	26.4	25.0	27.7	27.1	28.0	31.9	28.6	28.5
20	25.0	26.0	26.1	32.0	28.8	29.1	29.5	31.5	31.4	27.8
21	33.1	32.4	31.9	31.4	32.3	33.3	32.6	32.6	32.6	33.5
24	28.0	32.0	27.0	27.4	28.2	27.9	28.8	27.2	26.8	28.4
26	26.5	31.0	27.1	26.9	28.0	26.2	26.2	27.4	27.9	28.3
28	25.5	30.9	26.9	27.5	26.1	28.4	28.3	27.4	28.0	29.1
30	26.6	27.5	26.5	28.9	26.5	26.1	26.4	28.1	28.2	29.3

**Table 6:** Comparison of the SP-ISE and SC-ISE electrodes with the best selected parameters.

Ionophore + Plascticizer	SP-ISE	SC-ISE
none + NPOE	11-blank electrode	16- blank electrode
DBC + NPOE	13	18
HSB*β*CD + NPOE	14	19
none + DDP	12- blank electrode	17- blank electrode
HSB*β*CD + DDP	15	20
**Selection criteria**	**SP-ISE**	**SC-ISE**
Linear Range, pc	14, 15 3–6	18, 19, 20 3–6
Standard Deviation SD from E^0^, mV	14 35.12 15 17.70	18 7.16 19 4.36 20 9.04
Standard deviation from R^2^	14 0.0011 15 0.0005	18 0.0004 19 0.0009 20 0.0027
Dynamic Response	15 the most stable response at every concentration	20 the most stable response at every concentration
Reversibility of potential 10^−4^ mol L^−1^, SD, mV	14 1.05 15 1.42	18 0.54 20 0.71
Potential Drift	15 90 μV/min (120 min) 70 μV/min (240 min)	20 80 μV/min 50 μV/min
Selectivity, $K_{\frac{\mathrm{CHD}}{\mathrm{Acesulfame K}}}^{\mathrm{pot}}$	15 3.0 × 10^−6^	20 7.1 × 10^−7^
Water layer test	-	18 instability, possibility of a water layer 20 no water layer

**Table 7:** Results obtained in the analysis of chlorhexidine digluconate by the GC/MWCNT-s/ HSB*β*CD electrode No. 20 (the results are based on 3 measurements).

Sample.	Method	Taken mg L^−1^	Found mg L^−1^	Recovery [%]	Confidence Range, mg L^−1^
Pure (Alfa Aesar)	Calibration curve	89.78	90.7 ± 1.2	101.0	90.7 ± 3.1
	Standard Addition	89.78	91.6 ± 1.1	102.0	91.6 ± 2.6
	Gran’s method	89.78	89.3 ± 0.2	99.5	89.3 ± 0.3
Eludril (Pierre Fabre)	Calibration curve	199.31	205.5 ± 17.9	103.6	205.5 ± 44.3
	Standard Addition	199.31	191.2 ± 1.4	95.9	191.2 ± 3.4
	Gran’s method	199.31	196.6 ± 7.3	98.6	196.6 ± 18.0
HPLC [48] Cyteal Hexitol	Standard Addition	-	-	99.06 100.79	97.3–100.7% 96.8–104.7%

Confidence range $\mu \pm \frac{t}{\sqrt{n}}s,$ μ-mean, t-Student’s parameter, confidence level *p* = 95%.

**Table 8:** Potentiometric sensors for chlorhexidine comparison.

Parameter	Hussien, E.M. [43]	Magdy, N. [44]	Lenik, J. [42]	Present Work
Electrode configuration	Graphite disc	SPE	Ag/AgCl	GCE/MWCNT
Membrane	nail varnish + cellulose acetate propionate	PVC, DOP	PVC, DOS	PVC, DDP
Ionophore	CHD-tetraphenyl borate	2-HPβCD	HSBβCD	HSBβCD
Solid contact	not applicable	Gr Nc	not applicable	MWCNT
Slope, mV decade^−1^	29.0	28.8	30.4	29.4
E^0^ ± s, mV	not given	463.1	214.7 ± 40.6	539.36 ± 9.04
Linear range	10^−5^ ≥ 10^−3^	10^−6^–10^−3^	10^−6^–10^−3^	10^−6^–10^−3^
LOD, mol L^−1^	4 10^−6^	4.7 × 10^−7^	4.0 × 10^−7^	6.3 × 10^−7^
Water layer test	not applicable	performed, minimal water layer occurs	not applicable	performed, limited water layer formation
Potential stability, repeatability	Drift 1 mV/min in 10^−5^, 10^−4^, 10^−3^ mol L^−1^ solutions	Repeatability RSD = 0.57% in 10^−6^–10^−3^ mol L^−1^ solutions	Reversibility, standard deviation 3.3 mV in 10^−4^ mol L^−1^ solution	Reversibility, standard deviation 0.7 mV in 10^−4^ mol L^−1^ solution
Response time, s	Rapid no value provided	15	5	10
Real samples	pharmaceutical	pharmaceutical, human salvia	pharmaceutical	pharmaceutical

SPE—Carbon Screen-Printed; Gr Nc—graphene nanocomposite; 2-HPβCD—2-hydroxypropyl-β-cyclodextrin; DOP—dioctyl phthalate.

## Data Availability

Data are contained within the article.

## References

[1] Pawłowski M. (2020). Chemia Leków.

[2] McDonnell G., Russell A.D. (1999). Antiseptics and disinfectants: Activity, action, and resistance. Clin. Microbiol. Rev..

[3] Carrilho M.R., Carvalho R.M., Sousa E.N., Nicolau J., Breschi L., Mazzoni A., Tjäderhane L., Tay F.R., Agee K., Pashley D.H. (2010). Substantivity of chlorhexidine to human dentin. Dent. Mater..

[4] Agarwal A., Nelson T.B., Kierski P.R., Schurr M.J., Murphy C.J., Czuprynski C.J., McAnulty J.F., Abbott N.L. (2012). Polymeric multilayers that localize the release of chlorhexidine from biologic wound dressings. Biomaterials.

[5] Poppolo Deus F., Ouanounou A. (2022). Chlorhexidine in dentistry: Pharmacology, uses, and adverse effects. Int. Dent. J..

[6] Kathuria D., Raul A.D., Wanjari P., Bharatam P.V. (2021). Biguanides: Species with versatile therapeutic applications. Eur. J. Med. Chem..

[7] de Lima F.A., Tognolli M.F., Matos M.A.C., Nunes H.R., Nunes S. (2010). Analytical methods for the determination of chlorhexidine: A review. Crit. Rev. Anal. Chem..

[8] Szigeti Z., Vigassy T., Bakker E., Pretsch E. (2006). Approaches to improving the lower detection limit of polymeric membrane ion-selective electrodes. Electroanalysis.

[9] Radu A., Peper S., Bakker E., Diamond D. (2007). Guidelines for improving the lower detection limit of ion-selective electrodes: A systematic approach. Electroanalysis.

[10] Michalska A. (2012). All-solid-state ion selective and all-solid-state reference electrodes. Electroanalysis.

[11] Bobacka J. (2006). Conducting Polymer-Based Solid-State Ion-Selective Electrodes. Electroanalysis.

[12] Wardak C., Pietrzak K., Grabarczyk M. (2021). Ionic Liquid-Multiwalled Carbon Nanotubes Nanocomposite Based All Solid State Ion-Selective Electrode for the Determination of Copper in Water Samples. Water.

[13] Zhu J., Li X., Qin Y., Zhang Y. (2010). Single-piece solid-contact ion-selective electrodes with polymer–carbon nanotube composites. Sens. Actuators B Chem..

[14] Khaled E., Kamel M.S., Hassan H.N.A., Haroun A.A., Youssef A.M., Aboul-Enein H.Y. (2012). Novel multi walled carbon nanotubes/β-cyclodextrin based carbon paste electrode for flow injection potentiometric determination of piroxicam. Talanta.

[15] Bobacka J. (1999). Potential Stability of All-Solid-State Ion-Selective Electrodes Using Conducting Polymers as Ion-to-Electron Transducers. Anal. Chem..

[16] Yin T., Qin W. (2013). Applications of nanomaterials in potentiometric sensors. TrAC Trends Anal. Chem..

[17] Vázquez M., Bobacka J., Ivaska A., Lewenstam A. (2002). Influence of oxygen and carbon dioxide on the electrochemical stability of poly(3,4-ethylenedioxythiophene) used as ion-to-electron transducer in all-solid-state ion-selective electrodes. Sens. Actuators B Chem..

[18] Lewenstam A., Bobacka J., Ivaska A. (1994). Mechanism of ionic and redox sensitivity of p-type conducting polymers: Part 1. Theory. J. Electroanal. Chem..

[19] Bobacka J., Gao Z., Ivaska A., Lewenstam A. (1994). Mechanism of ionic and redox sensitivity of p-type conducting polymers: Part 2. Experimental study of polypyrrole. J. Electroanal. Chem..

[20] Bobacka J., Ivaska A., Lewenstam A. (2003). Potentiometric Ion Sensors Based on Conducting Polymers. Electroanalysis.

[21] Imali D.Y., Perera E.C.J., Kaumal M.N., Dissanayake D.P. (2024). Conducting Polymer Functionalization in Search of Advanced Materials in Ionometry: Ion-Selective Electrodes and Optodes. RSC Adv..

[22] Gao L., Tian Y., Gao W., Xu G. (2024). Recent Developments and Challenges in Solid-Contact Ion-Selective Electrodes. Sensors.

[23] Bomar E.M., Owens G.S., Murray G.M. (2017). Nitrate Ion Selective Electrode Based on Ion Imprinted Poly(N-Methylpyrrole). Chemosensors.

[24] Mu S., Yin T., Luan F., Qin W. (2024). High-Redox-Capacity Solid Contact Based on Ferrocenyl Self-Assembled Monolayer Functionalized Macroporous Gold for an All-Solid-State Carbonate-Selective Electrode. Microchem. J..

[25] Rousseau C.R., Honig M.L., Bühlmann P. (2022). Hydrogels Doped with Redox Buffers as Transducers for Ion-Selective Electrodes. Anal. Chem..

[26] Morawska K., Wardak C. (2024). Application of Ionic Liquids in Ion-Selective Electrodes and Reference Electrodes: A Review. ChemPhysChem.

[27] Mirabootalebi S.O., Liu Y. (2024). Recent Advances in Nanomaterial-Based Solid-Contact Ion-Selective Electrodes. Analyst.

[28] Zeng X., Yu S., Yuan Q., Qin W. (2016). Solid-Contact K^+^-Selective Electrode Based on Three-Dimensional Molybdenum Sulfide Nanoflowers as Ion-to-Electron Transducer. Sens. Actuators B Chem..

[29] Lai C.-Z., Fierke M.A., Stein A., Bühlmann P. (2007). Ion-Selective Electrodes with Three-Dimensionally Ordered Macroporous Carbon as the Solid Contact. Anal. Chem..

[30] Huczko A. (2004). Nanorurki Węglowe: Czarne Diamenty XXI Wieku.

[31] Parra E.J., Crespo G.A., Riu J., Ruiz A., Rius F.X. (2009). Ion-Selective Electrodes Using Multi-Walled Carbon Nanotubes as Ion-to-Electron Transducers for the Detection of Perchlorate. Analyst.

[32] Porto L.S., Silva D.N., de Oliveira A.E.F., Pereira A.C., Borges K.B. (2020). Carbon Nanomaterials: Synthesis and Applications to Development of Electrochemical Sensors in Determination of Drugs and Compounds of Clinical Interest. Rev. Anal. Chem..

[33] Sacramento A.S., Moreira F.T.C., Guerreiro J.L., Kamel A.H., Sales M.G.F. (2017). Novel Biomimetic Composite Material for Potentiometric Screening of Acetylcholine, a Neurotransmitter in Alzheimer’s Disease. Mater. Sci. Eng. C.

[34] Khalil M.M., El Rouby W.M.A., Korany M.A. (2019). Potentiometric Sensor Based on Novel Flowered-Like Mg-Al Layered Double Hydroxides/Multiwalled Carbon Nanotubes Nanocomposite for Bambuterol Hydrochloride Determination. Mater. Sci. Eng. C.

[35] Lenik J., Nieszporek J. (2018). Construction of a Glassy Carbon Ibuprofen Electrode Modified with Multi-Walled Carbon Nanotubes and Cyclodextrins. Sens. Actuators B Chem..

[36] Kammoun A.K., Abdelrahman M.H., Khayyat A.N., Elbaramawi S.S., Ibrahim T.S., Abdallah N.A. (2023). Exploitation of Multi-Walled Carbon Nanotubes/Cu(II)-Metal Organic Framework Based Glassy Carbon Electrode for the Determination of Orphenadrine Citrate. RSC Adv..

[37] Tantawy M.A., El Fiky H.A., Badawey A.M., Abd El Ghany M.F., Fares N.V. (2021). A Novel Glassy Carbon Electrode Modified with Multi-Walled Carbon Nanotubes for Potentiometric Xipamide Determination. J. Electrochem. Soc..

[38] Hussein O.G., Monir H.H., Zaazaa H.E., Galal M.M. (2024). Eco-Conscious Potentiometric Sensing: A Multiwalled Carbon Nanotube-Based Platform for Tulathromycin Monitoring in Livestock Products. BMC Chem..

[39] Borg H., Belal F., Draz M.E. (2022). Facile fabrication of a portable PANI-NFs/c-MWCNT nano-composite electrochemical sensor for gefitinib: Application to human plasma. Anal. Methods.

[40] Wardak C., Pietrzak K., Morawska K., Grabarczyk M. (2023). Ion-Selective Electrodes with Solid Contact Based on Composite Materials: A Review. Sensors.

[41] Huang M.-R., Li X.-G. (2022). Highly sensing and transducing materials for potentiometric ion sensors with versatile applicability. Prog. Mater. Sci..

[42] Lenik J., Sokal K. (2023). A Novel Polymeric Membrane Sensor for Chlorhexidine Determination. Sensors.

[43] Hussien E.M. (2014). Novel PVC-Free All-Solid-State Ion-Selective Electrode for Determination of Chlorhexidine in Pharmaceutical Formulations. Anal. Bioanal. Electrochem..

[44] Magdy N., Sobaih A.E., Hussein L.A., Mahmoud A.M. (2022). Graphene-based disposable electrochemical sensor for chlorhexidine determination. Electroanalysis.

[45] Bakker E., Pretsch E., Bühlmann P. (2000). Selectivity of Potentiometric Ion Sensors. Anal. Chem..

[46] Pampani P., Shenoy S., Anegundi R.V., Shekar M.K., Dharani K., Fathima L. (2024). Estimation of Salivary Sodium, Potassium, and Calcium Levels in Periodontal Health and Disease. J. Indian Soc. Periodontol..

[47] Fibbioli M., Morf W.E., Badertscher M., de Rooij N.F., Pretsch E. (2000). Potential Drifts of Solid-Contacted Ion-Selective Electrodes Due to Zero-Current Ion Fluxes Through the Sensor Membrane. Electroanalysis.

[48] Sobaih A.E., Magdy N., Hussein L.A. (2022). HPLC Method for Determination of Chlorhexidine in Pharmaceutical Formulations alone and in presence of Hexamidine and p-chlorocresol, and in Spiked Human Saliva. Arch. Pharm. Sci. Ain Shams Univ..

